# The impact of neoliberal generative mechanisms on Indigenous health: a critical realist scoping review

**DOI:** 10.1186/s12992-022-00852-2

**Published:** 2022-06-15

**Authors:** Brianna Poirier, Sneha Sethi, Dandara Haag, Joanne Hedges, Lisa Jamieson

**Affiliations:** Australian Research Centre for Population Oral Health, Adelaide Dental School, University of Adelaide, Adelaide, SA 5005 Australia

**Keywords:** Neoliberalism, Indigenous health, Health equity, Globalisation, Indigenous peoples, Critical realism

## Abstract

The pervasive nature and colonial foundations of neoliberalism has significant ramifications for Indigenous health, globally. Not only does neoliberalism undermine Indigenous collectivist values by emphasising personal autonomy, but the exploitation of natural resources has unique implications for Indigenous wellbeing. Therefore, this scoping review aims to synthesise evidence that articulates the impacts of neoliberalism on global Indigenous health inequities. Two reviewers searched PubMed, Embase, Scopus, Web of Science, and ProQuest Central to identify records eligible for inclusion. The search was not restricted by geographic location or language. Using principles of qualitative meta-aggregation, generative mechanism summaries and illustrations were extracted from each of the included articles, synthesised into broader categories, then considered in the context of neoliberal ideologies. The systematic search identified 9952 unique records, of which 38 fully satisfied the inclusion criteria. Findings represented 23 Indigenous communities across 12 countries and considered the impacts of neoliberalism across 16 health outcomes. Eighty-eight generative mechanisms of neoliberalism and 12 generative mechanisms of resistance were extracted from the included articles and mapped against four core principles of neoliberalism: competitive and private markets, reduced public expenditure on infrastructure, personal autonomy, and deregulation that facilitates economic activity. Overwhelmingly, neoliberalism has manifest impacts, through various pathways, on poor health outcomes and experiences for Indigenous communities included in this review. Importantly, Indigenous communities continue to resist the impacts of neoliberalism through advocacy, reclamation of traditional practices, and opposition to industrial development. Consideration and investigation of neoliberal structures and ideologies must become common practice in health equity scholarship. Actors within neoliberal societies must resist dominant epistemological, ontological, and praxiological stances that reinforce the supremacy of colonial values and subalternation of Indigenous ways of knowing, being, and doing to begin effectively addressing Indigenous health inequities.

**Supplementary Information:**

The online version contains supplementary material available at 10.1186/s12992-022-00852-2.

## Introduction

The wealth of linguistic, cultural, and knowledge diversity of the 400 million Indigenous peoples globally represents an invaluable resource for human development (1). The United Nations considers Indigenous peoples as those with historical continuity and pre-colonial societies whom consider themselves distinct from societies now residing on their ancestral lands; this definition also emphasises that presently, Indigenous communities form non-dominant sectors of society yet remain determined to preserve ancestral territories and transmit knowledges to future generations (1). While various international and national policies and frameworks assert the need for Indigenous health equity, these inequities persist and, in some cases, have worsened (2). Indigenous people remain on the periphery of dominant cultures and societies, bearing an inordinate burden of disease, poverty, and mortality compared to non-Indigenous populations (3, 4). The social determinants of health framework (5) in combination with the recognition of the implications of colonisation and assimilation policies have enriched understandings of the complexities of Indigenous health inequities (6). This perspective includes structural and sociopolitical factors that contribute to the intersecting axes of oppression, including racism, environmental dispossession, poverty, as well as social and political exclusion that determine the health of Indigenous peoples (7–9). The pervasive and damaging impacts of neoliberal policies and ideologies have been recognised as having impacted the lives of a large majority of Indigenous peoples to an increasingly intolerable extent (10).

Neoliberalism gained traction as a set of dominating ideologies, practices, and policies during the Cold War period and consequently underpinned the globalisation movement. Championed by economists Friedman and Hayek, and politicians Reagan and Thatcher, neoliberalism prevails today as the dominant global political orientation (11, 12). The core principles of neoliberalism include personal autonomy, competitive private markets, reduced public expenditure on infrastructure, health and social services, and deregulation that supports the free market and economic activity (13, 14). Despite the widespread claim of economic and social triumph among advocates of neoliberalism, this ‘victory’ has come at a great cost to societies, particularly for those experiencing various forms of social disadvantage (15). Neoliberalism’s ubiquitous impact on human lives operates within increasingly coercive and detrimental power frameworks (15). Regrettably, circumstances of power imbalances are a common experience for Indigenous communities globally, who have shared histories of imposed policies of assimilation, dispossession, and oppression (6, 8). Experiences of historic trauma and institutional racism exemplify non-Indigenous power over Indigenous peoples, and present-day ideologies of neoliberalism facilitate the continuation of oppressive circumstances and the maintenance of health inequities between Indigenous and non-Indigenous populations (16).

The reporting of health inequities between Indigenous and non-Indigenous peoples has mirrored the neoliberal ideology of personal autonomy for some time, furthering deficit discourses (17) that attempt to justify the significant health disparities experienced by many Indigenous communities. This insufficient understanding of Indigenous health arguably causes more harm to Indigenous peoples and limits opportunities and resources for substantial improvements in health outcomes (15, 18, 19). Policies of colonisation and assimilation restricted personal control over the lives of many Indigenous people; the shift to a neoliberal discourse of personal autonomy, which blames individuals for their circumstances and behaviours, largely ignores the vast impact of previous political circumstances on Indigenous wellbeing (14, 20). Beyond considerations of assimilation and colonisation, attention to modern colonial values, political economies, and structural factors that limit individual choice for Indigenous peoples is needed. The forced operation of Indigenous peoples within socio-political structures of dominant culture replicates power frameworks established during colonisation (21). Therefore, scholars, politicians and policy makers must move beyond the acknowledgment of historic oppression and develop a more nuanced consideration of how modern-day structural forces maintain and strengthen power imbalances and contribute to Indigenous health inequities. The investigation of Indigenous health which considers historical socio-political circumstances permits a shift away from personal responsibility for health and creates space to explore alternative pathways and interventions to achieving Indigenous health equity. This aligns with a strength-based approach rather than continuing a deficit discourse, which disempowers Indigenous communities (17).

The emphasis on personal autonomy and individualism in neoliberal discourse contradicts widely shared Indigenous collectivist values that facilitate strong social cohesion (20). Dominant culture poses a threat to Indigenous cultures in that neoliberal values diminish social cohesion and increase social divides; failing to challenge these accepted ways risks normalising individualism and isolation (19). Investigating the impact of neoliberalism on Indigenous health through the utilisation of Indigenous experiences provides a unique perspective, from the periphery of dominant culture, that can facilitate a shift in the perceptions of individuals and systems embedded in neoliberalism’s hegemony (22). Despite emerging literature investigating the impacts of neoliberalism in various areas of Indigenous health, there remains limited conceptual understandings of the ways in which neoliberalism broadly influences Indigenous health and wellbeing. This information is essential for producing evidence needed to inform interventions aimed at counteracting the negative impacts of neoliberal practices on the health of Indigenous populations. Therefore, this scoping review seeks to identify and synthesise generative mechanisms which articulate the impacts of neoliberalism on Indigenous health, globally.

## Materials and methods

Scoping reviews are a specific type of systematic literature review that aim to identify all available literature on a specified topic, regardless of methodological rigour, often including grey literature sources (23). An initial search of the International Prospective Register of Systematic Reviews, PubMed, and the Joanna Briggs Systematic Reviews register revealed no similar published or underway studies. This scoping review has been registered with the Joanna Briggs Systematic Reviews register, and in accordance with Joanna Briggs Institute methodological recommendations for scoping (23), the protocol was made publicly available with the Center for Open Science (24). This review was conducted and reported in alignment with the Preferred Reporting Items for Systematic Reviews and Meta-Analyses guidelines (Supplemental File 1).

### Positionality

Qualitative research largely rejects ideas of emergence and objectivity, and instead embraces the influence of researcher subjectivity on findings. Accordingly, acknowledging the positionality and influence of those involved in the research process is essential (25). As privileged, educated, female researchers, the review team (BP, SS, DH) and senior author (LJ) have benefitted from processes of neoliberalism throughout their lives and will never entirely understand the influence of neoliberalism on Indigenous health. Therefore, the involvement of a senior Indigenous researcher (JH) in this review was critical to maintaining relational understanding of stories collated during this research process.

### Theoretical foundations

This review was conducted from the metatheoretical perspective of critical realism (26, 27), which understands social phenomenon as consisting of and constituted by different layers of reality, where causality must be explored on layers beneath the observable and empirical (28). Critical realism aims to elicit transformative change through the identification and comprehension of the contingencies that evoke causal pathways for a given outcome (27), which for this research is inequitable experiences of health among global Indigenous communities. Determining aspects related to the outcome of interest creates an opportunity to categorise social complexities of actions, occurrences, and decisions, that work to form underlying structures of causal power, also known as generative mechanisms (27, 28). From the perspective of critical realism, generative mechanisms penetrate the empirical surface and forge contact with reality that exists beneath the level of observable events. While generative mechanisms exist in the social world they are regarded as tendential, or not observable in empirical events, and require auspicious conditions. We must be cognisant that mechanisms can contradict and contrast each other and while existent, certain contexts can prevent observable effects from taking place; in an empirical correlation study, this may lead to a conclusion of no effect. However, critical realism postulates that it is possible to draw conclusions about the interaction of existing generative mechanisms even in circumstances that prevent observable effects (28). In line with the socially focused aspects of critical realism, the methodological approach to this review was informed by decolonising theories (29, 30). Decolonising theories highlight the impacts of ongoing colonisation and the related marginalisation from dominant culture, where dominant culture is understood to be aligned with neoliberal and colonial values.

A Western theoretical framework of neoliberalism was utilised in this review to enable a critique of the Western understandings of neoliberalism considered in the articles included in this review. Four domains of neoliberalism were considered in this review: competitive and private markets, reduced public expenditure on infrastructure and social services, personal autonomy, or deregulation that facilitates economic activity (13). Importantly, generative mechanisms in this review do not ascertain a direct ‘generation’ of impact from neoliberalism itself, but are understood as expressions of the general phenomenon of neoliberalism (27, 28).

### Identifying articles for inclusion

Six databases were searched in August 2021 using keywords and index terms related to “neoliberalism,” “Indigenous,” and “health.” The search strategy was first developed for PubMed and then adapted as per the design of Embase, Scopus, Web of Science, and ProQuest Central (Supplemental File 2). The search was not restricted by language, study design, or geographic location. The search was restricted to articles published from January 1 1975, until the search date to capture effects of modern conceptualisations of neoliberalism, which gained popularity with the election of Ronald Reagan (United States) and Margaret Thatcher (United Kingdom) in the late 1970s (31). For the purposes of this review, articles that discussed generative mechanisms of either neoliberalism or globalisation described in accordance with the Oxford Dictionary definition of “a political approach that favours free-market capitalism, deregulation, and reduction in government spending,” (32) in relation to Indigenous health, defined as “more than just the absence of disease or illness; it is a holistic concept that includes physical, social, emotional, cultural, spiritual and ecological wellbeing, for both the individual and the community,” (33) were considered for inclusion.

After the removal of duplicates, two independent reviewers (BP, SS) screened the titles and abstracts of articles identified in the systematic search in Endnote (Clarivate Analytics, PA, USA), with articles considered relevant by either reviewer progressing to full-text review. Full-text articles were subsequently screened against the inclusion criteria (Table 1), by the same reviewers. Eight of the papers identified in the systematic search were in Spanish, so a third reviewer (DH) fluent in Spanish performed full-text screening and data extraction for these articles to minimise loss of meaning and other potential errors associated with translating these articles to English. Any disagreements or uncertainties were resolved through discussion among the three reviewers. In accordance with scoping review methodologies, critical appraisal was not performed on studies included in this review because the aim was not to produce critically appraised findings but to provide an overview of existing evidence (23).

**Table 1:** Inclusion and exclusion criteria

Inclusion	Exclusion
• Indigenous population (1) • The impact of neoliberalism (13, 32) (or ‘globalisation’) discussed in relation to an Indigenous health (33) outcome • All languages • All locations • Participants of all ages and genders	• Published before 1 January 1975 • Non-Indigenous population • Does not explicitly discuss the impact of neoliberalism on a health inequity or outcome

### Data extraction and synthesis

Data were compiled into a piloted extraction form by three reviewers (BP, SS, DH). Three articles were performed by all reviewers to ensure inter-rater reliability and to reduce the introduction of selection bias (34) of generative mechanisms within articles. The data extracted included information about participants, study aim, context, methods, key findings, generative mechanism summaries and associated illustrations from each article. Standard characteristics of each article were collated into a table. Data regarding geographic location and health inequity considered were tabulated and synthesised in a visual representation of geographic spread. The different neoliberal terminologies employed by authors of included articles were compared and narratively synthesised.

The synthesis approach utilised in this scoping review borrowed heavily from the Joanna Briggs Institute methodological guidelines for meta-aggregations in qualitative systematic reviews (35). A qualitative systematic review was not appropriate for this review because many of the generative mechanisms were not substantiated with empirical evidence and participant illustrations, as is needed for a meta-aggregation. Three reviewers (BP, SS, DH) comprehensively reviewed included articles line-by-line to extract generative mechanisms of neoliberalism. Exact wording of each mechanism was extracted, and each reviewer created a one line ‘generative mechanism summary’ that captured the essence of the extracted evidence, similar to the themes extracted with findings in a meta-aggregation (35). These findings were then compiled in Microsoft Excel. Unlike a meta-aggregation, findings were not scored for credibility, because all sources of evidence were considered in this systematic scoping review (23). In the initial phases of extraction, reviewers observed the description of resistance against neoliberal forces impacting Indigenous health. This led to the decision to also extract generative mechanisms of resistance to ensure the presentation of a more comprehensive view of Indigenous communities impacted by neoliberal ideologies. The few articles for which extraction had been completed were revisited to ensure all generative mechanisms of resistance were identified. The synthesis of evidence was done manually by the review team, which involved writing all generative mechanism summaries on a whiteboard and identifying common concepts across the data. Common generative mechanisms were then considered in the context of four core neoliberal principles and mapped to one of: competitive and private markets, reduced public expenditure on infrastructure and social services, personal autonomy, or deregulation that facilitates economic activity.

## Results

**Fig. 1:**
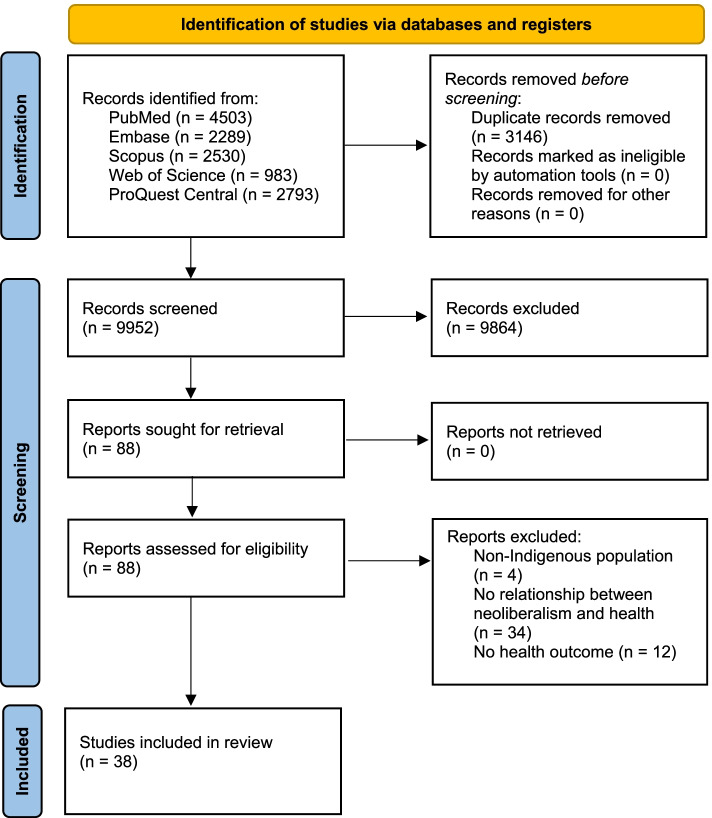
PRISMA 2020 flow diagram

The systematic search located 13,098 articles, of which 3146 were duplicates, leaving 9952 articles eligible for inclusion in this scoping review. After title and abstract screening, 88 articles were retrieved and assessed for eligibility against the inclusion criteria during full-text review. Fifty articles were deemed ineligible during this process, primarily due to a lack of generative mechanism identified by authors that detailed the relationship between neoliberalism and an Indigenous health inequity or outcome. Therefore, a total of 38 articles were included in this systematic scoping review (Fig. 1).

### Study characteristics

The included articles were published between 1983 and 2021. Ten articles were from Canada (36–45), five from Australia (46–50), four from Aotearoa/New Zealand (51–54), three from the United States (55–57), two from Guatemala (58, 59) and Brazil (60, 61) and one article from each of Venezuela (62), Ecuador (63), Bolivia (64), Chile (65), Mexico (66), and Peru (67). Six of the included articles had a global or multiple country focus (68–73). Papers discussed health concerns among Ojibway (36), Cree (36), Namg (38), Dene (39), Mi’kmaq (41), Haisla (43), First Nations (37, 42, 44, 45, 71), Inuit (37, 40, 45, 69), Métis (37, 45), Sámi (69, 71), Māori (51–54, 71), Aboriginal and Torres Strait Islander (46–50, 71), American Indian (56, 71), Alaskan Native (57, 71), Warao (62), Kichwa (63), Tsimane (72), Huaorani (72), Mayan (59, 66), Guarani (61), Kaingang (61), Williche (65), Mbyá-Guaraní (73), Chimanes (73), Moxeños (73), Yuracarés (73), Peruvian (67), and Amazonian (64) Indigenous communities. Included articles ranged in study design; eight commentaries (41, 47, 51, 62, 66, 70, 71, 73), seven reviews (36, 40, 46, 52, 68, 69, 72), seven qualitative studies (38, 50, 53–55, 61, 63), four news articles (49, 56, 59, 64), three dissertations (39, 57, 58), three essays (42, 44, 67), two mixed methods studies (45, 60), two case studies (48, 65), one book chapter (43), and one editorial (37) were included in this review (Supplemental File 3).

### Synthesis of evidence

The term ‘neoliberalism’ is used in academic literature to convey various meanings. To that end, the review team sought to identify how authors of included studies used the term neoliberalism in their papers. Twenty-four of the included studies did not explicitly define neoliberalism in relation to their work (36–39, 42, 43, 46, 49, 52–56, 59–61, 63–65, 67–69, 72, 73). Eight (40, 45, 48, 50, 57, 62, 70, 71) of the remaining 17 papers provided a comprehensive definition that considered the four core principles of neoliberalism used to frame this scoping review; competitive and private markets, reduced public expenditure, personal autonomy, and deregulation that facilitates economic activity. Five papers (40, 47, 58, 62, 66) warned about the threat of endangered livelihoods that neoliberalism poses for some, particularly Indigenous communities already experiencing social disadvantage, with one paper citing neoliberalism as a “leap backwards to a civilisation: ‘locked in the grip of an ideology’” (47). Five papers also emphasised the “cultural values of radical individualism” (50) championed by neoliberal ideologies (44, 47, 48, 50, 57). Three papers referenced the University of Chicago (Friedman & Hayek) origin of neoliberalism (57, 70, 71) and three papers also noted that neoliberalism was driven by wealthy OECD (Organisation for Economic Co-operation and Development) countries (62, 70, 71).

**Fig. 2:**
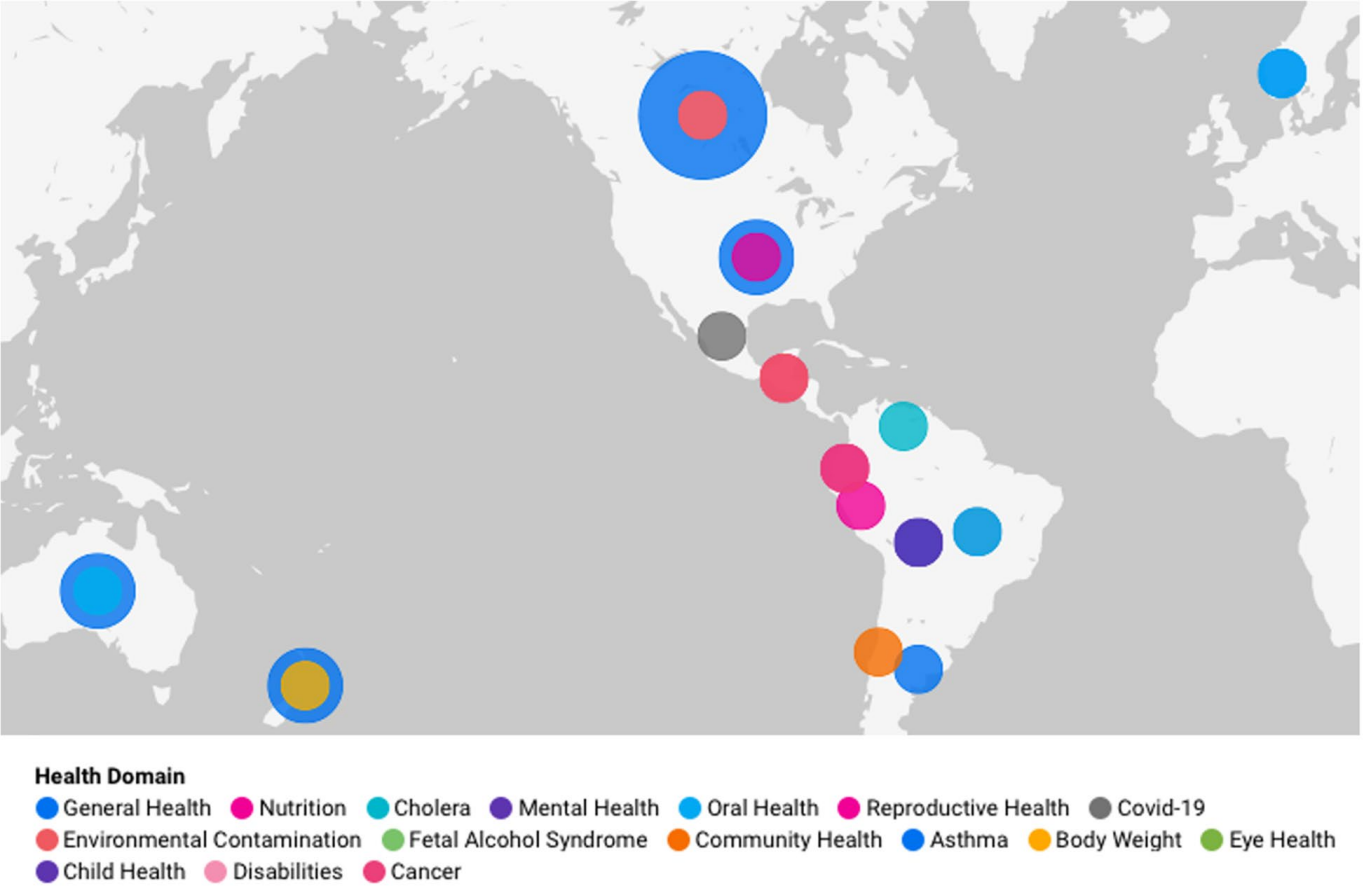
Geographic spread of included papers by health domain. Note: The size of each bubble corresponds to the number of papers from a given country and the colour corresponds with the health domain

The papers included in this review considered the impacts of neoliberalism on a variety of health domains. Fourteen of the included papers discussed multiple health outcomes or Indigenous health generally (37–40, 49, 51–53, 56, 57, 68, 73), four focused on oral health (46, 61, 70, 71), three examined nutrition (41, 58, 72), two explored mental health (45, 47), reproductive health (55, 67), environmental contamination (36, 59), and Covid-19 (60, 66), while there was one paper focused on asthma (43), body weight (54), cancer (63), child health (64), cholera (62), community health (65), disabilities (44), eye health (50), and foetal alcohol syndrome (42) respectively. The spread of health domains distributed geographically is visually represented in Fig. 2.

### Generative mechanisms synthesis

**Fig. 3:**
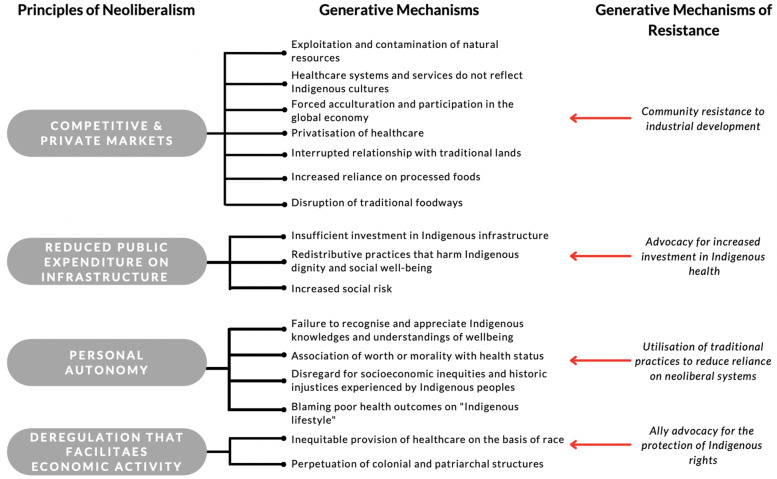
Conceptual model of the relationship between principles of neoliberalism and generative mechanisms

One hundred generative mechanisms were extracted from the articles included in this systematic scoping review, 12 of which were considered generative mechanisms of resistance against forces of neoliberalism impacting Indigenous health (Supplemental File 4). These generative mechanisms were synthesised into twenty overarching generative mechanisms, which were then mapped against four core principles of neoliberalism: competitive and private markets, reduced public expenditure, personal autonomy, and deregulation that facilitates economic activity (Fig. 3). It is important to note that several generative mechanisms are related to more than one principle of neoliberalism; for the purposes of this review, generative mechanisms were mapped to the core principle that most aligned with the original presentation of evidence.

### Impacts of neoliberal generative mechanisms on indigenous health

#### Competitive and private markets

Seven overarching generative mechanisms related to the influence of competitive and private markets on Indigenous health inequities. *Exploitation and contamination of natural resources* largely related to the contamination of traditional foods and resources, including water, due to industrial development (36, 39, 40, 55, 59, 63, 73). In Ecuador, increased occurrence of cancer among Kichwa communities was attributed to the use of chemicals, hormones, and antibiotics to increase crop yields and subsequently, market uptake, as one participant detailed, “*Like I was saying, they put them so they grow faster and now they fumigate and add more, and then everything grows really quickly and that is also causing a lot of harm to people I think”* (63). Similarly, a Native American community in the United States likened the rise in chronic health conditions due to industrial pollution to a modern-day genocide of their people, *“You have all of this industrial stuff… We did not have that when we were young… I think that is why our people have cancer. They were not used to this…This is what will kill… I said, ‘John Wayne did not live long enough to kill all of the Indians, so that’s what they want to do, finish killing the Indians’”* (55). Water contamination was also expressed as a concern by Indigenous communities in Canada, Guatemala, Bolivia, and Argentina, relating to environmental contamination, in terms of polluted drinking water and aquatic food sources (39, 40, 59, 73). For Ojibway and Cree First Nations in Canada, high levels of poisonous substances in the water led to a ban on fishing which had significant impacts on the economic, social status, and health of Indigenous communities (36). Exploitation of natural resources due to industrialisation saw deforestation, monocultures, and irreversible damage to previously flourishing ecosystems for Indigenous communities in South America (73).

The push for increased industrialisation due to competitive and private markets has *interrupted relationships with traditional lands* and subsequently impacted health outcomes for Indigenous communities in Canada (36, 38, 39), the United States (57), Bolivia (73), and Argentina (73). The feeling associated with the loss of traditional lands and foods for Inuit communities has been termed, “lonely for the ice,” (36) while Alaskan Natives refer to this feeling as a “soul wound” (57). This is illustrated by an Inuit Elder’s reflection on her ‘loneliness for the ice,’ *“I wish I could turn the clock back. I wish. What I see today I wish I could turn the clock back [on] all the mining that you know that oil spill, the oil, and all over our country you know. I don’t know if things are ever going to change back. It’ll take a long time.”* This feeling of loss has manifested as asocial behaviours among Ojibway and Cree First Nations (36) and poor health for Namg First Nations, which is attributed to the severing of spiritual connections (38). In Bolivia, Argentina, and Alaska interrupted relationships with traditional lands has resulted in increased drug and alcohol use, and suicide rates (57, 73).

The impact of *increased reliance on processed foods* was documented among Indigenous communities in Canada (36, 39, 71), Ecuador (63, 72), Australia (46, 71), the United States (55, 71), Guatemala (58), Bolivia (72), Aotearoa/New Zealand (71), and Norway (71). The inability of Indigenous peoples to depend on traditional foods due to factors including environmental contamination or participation in the economy (which reduces available time to take part in food sovereignty practices) has resulted in an increased reliance on processed, often termed ‘Western’, foods. Western foods were described by authors as calorie dense, nutrient poor, and unhealthy (39, 55, 58); a direct contrast to traditional diets, which are low in sugar and high in protein and nutrients. Harvesting traditional foods also contributes to physical fitness (39). Ojibway and Cree First Nations in Canada refer to traditional foods as a pure form of “genuine foods,” whereas Western foods were deemed altered or poisoned forms of foods (36). Contamination of waterways has similarly influenced beverage choices among Indigenous communities in Aotearoa/New Zealand, Australia, Canada, and the United States; drinking water has been replaced by sugar sweetened beverages due to their availability and affordability (70, 71). Kichwa community members in Ecuador have attributed the rise in cancer among their community to poor nutrition (“mala alimentacion”) associated with the increased preference for Western foods over traditional home cooked meals (63). Three authorship teams highlighted the impact of intense marketing of Western foods, particularly sugar, on Indigenous consumption and consequently, oral diseases (46, 70, 71). These factors, paralleled with increased availability and affordability of Western foods, has created increased susceptibility to diet-related chronic diseases, including cardiovascular disease, cancer, obesity, and type 2 diabetes (39, 55, 63, 72). While reliance on Western foods in many instances was a coping mechanism to overcome the impacts of industrial pollution and environmental contamination resulting from neoliberal ideologies, this transition has serious implications for Indigenous health (39, 58).

The means through which neoliberalism *disrupts traditional foodways* and impacts Indigenous health outcomes was detailed by research among Canadian (39, 41), Brazilian (61), and Arctic Indigenous communities (69). Dene First Nation members described the implications of increased mining on their traditional lands for local caribou, *“The way you look at it now, all the mines started it. All the animals we traditionally hunted are all gone now. Just all mines now. All the animals are making different routes now. Before I was used to having lots of caribou before all that mine stuff. Caribou not as much now… There’s another mine they want to open and in our hunting area. The moose are there. When that mine starts there’s going to be nothing because all the animals will move, will migrate again with all the blasting”* (39). In addition to impacts on those directly involved in hunting, disruption of traditional foodways has manifest ramifications on lifestyle practises, with many Indigenous communities having extensive food sharing networks that are important for nutrition as well as maintaining and strengthening cultural connections. (69). Similar to Dene First Nation, implications of climate change among Arctic communities, including melting ice and thawing permafrost, are effectively severing community reliance on traditional wildlife due to the degraded quality of local foods. Additionally, permafrost ice houses, typically used by Arctic Indigenous communities to store traditional foods, are presenting food contamination challenges (69). Governance models framed under the guise of reconciliation target land-based practices of Indigenous communities in Canada (41) and restrict access to traditional lands for Indigenous communities in Brazil, constituting a substantial threat to Indigenous food sovereignty while simultaneously increasing monetary dependence of communities (61).

The impact of *privatisation of healthcare* was discussed in relation to Māori (51), Aboriginal and Torres Strait Islander (46), and Mayan (66) Indigenous health. Barnett and Bagshaw highlighted the false claims of health services overfunding made in the 1980s by the Aotearoa/New Zealand government and reinforced by self-interested private organisations; health expenditure modelling of Treasury models from that time revealed a cumulative decline in health spending (51). Unfortunately, this neoliberal narrative effectively reduced funding for health services which supported Māori wellbeing. Due to the privatisation of dental services in Australia, use of services for many Aboriginal and Torres Strait Islander has been constrained to treatment focused care rather than preventive care due to associated financial costs of both private and public services, which often incur a co-payment (46). In Mexico, the commonality of understocked and intermittently staffed community clinics recently saw an increase in susceptibility to workforce and material shortages due to Covid-19 recruitment of medical personnel to hospitals; this left Mayan communities with extremely limited access to healthcare of any kind (66).

In addition to implications of privatisation, many *healthcare systems and services fail to reflect Indigenous cultures*. Both commodification of care and biomedical neoliberal language was highlighted as impacting Indigenous community health in Aotearoa/New Zealand (51), Australia (50), and Canada (38, 44). For some Aboriginal and Torres Strait Islander communities with low fundamental English literacy, individuals described biomedical language as confusing and frightening, leading to communication breakdowns, and ultimately, exclusion from eye health knowledge and decision making, *“The doctor they frighten you too, it’s how they talk you know?”* (50). Similarly, an Elder from Namg First Nation in Canada described the connection between traditional languages and health and the impact of dispossession, as a result of biomedical neoliberal language, on mental and spiritual health, *“Our language is our culture; it is the medium, or the form, or the process, that allows us to give full expression to who we are, mentally, physically, spiritually, collectively, as friends and family, individually, historically [and] looking forward. It’s the only medium we have that can do that. As long as we have our mind-set we’re not going to be struggling with Western concepts [like] what’s right or wrong. The creator never intended that to be the way it is. We’re Kwakwaka’wakw and he gave us laws that are spiritual, that will sustain us through time. We will be the healthiest when we can give expression to that”* (38). Indigenous families in Canada identify a gap in provision of care related to the government approaches to disability and childhood, which translates into reliance on commodification of care, paying for services of professionals and failing to recognise the value of a mother’s knowing and care for her children (44).

Finally, the expansion of private and competitive markets has resulted in a *forced acculturation and participation in the global economy* for many Indigenous communities (68), including those in Ecuador (63), Bolivia (73), Argentina (73), Canada (39), Mexico (66), and Brazil (61). Four of the included articles discussed the implications of the transition from traditional economies to participation in the wage work economy; many Indigenous families in these articles were required to move to bigger cities, leaving home, and increasing consumption of Western foods (39, 61, 63, 66). Kichwa communities in Ecuador attributed the rise in cancer to participation in the global economy with many women now working outside of the home. Both men and women described their reliance on public transportation to travel to work instead of walking; this combined with decreased free time and increased purchasing power associated with a dual income household resulted in higher consumption of Western foods (63). Similarly, Dene First Nation in Canada are increasingly partaking in wage work which requires employment Monday through Friday, limiting time for Indigenous peoples to partake in land-based and harvesting activities; this further compounds the widespread loss of traditional skills and knowledges due to the impacts of Residential Schools (39). Similar sentiments were echoed by the Kaingang in Brazil, who attribute the recent changes in eating practices to lifestyle changes due to participation in the formal labour market, which is effectively discontinuing practices of food cultivation among youth and has implications for the emotional importance of traditional foods, which are now only reserved for special occasions (61). Many Mayan individuals in Mexico work in the cruise ship and tourism business, which resulted in increased transmission and early exposure to Covid-19; work continued for a month until public health measures were implemented and Mayan people returned to their communities, bringing Covid-19 with them (66). The reliance on translators in Bolivia and Argentina has disrupted and destabilised Indigenous communities, resulting in a “crisis of representation” where non-leaders, who are in need of money, are utilised by government or outsiders to gain access to community knowledges (73). The integration of Indigenous peoples in the global economy often occurs without appreciation for self-determination and associated social and political violence. Acculturation has been linked to issues of addiction, suicide, and weakened social networks and knowledge systems, which are fundamental to Indigenous health and adaptive capacity (68).

#### Reduced public expenditure on infrastructure

Three overarching generative mechanisms related to the influence of reduced public expenditure on Indigenous health outcomes. *Insufficient investment in Indigenous infrastructure* was highlighted in Canada (37, 41, 43, 45, 71), Chile (65), Australia (50, 71), Aotearoa/New Zealand (71), and the United States (71). The need to ‘close the gap’ between Indigenous and non-Indigenous health, touted by many Indigenous and non-Indigenous leaders, is directly contradicted by the neoliberal funding models these leaders operate within. In Canada for First Nations, Inuit, and Métis communities this contradictory unequal distribution of social programs is accelerating health and social inequities (37) and manifesting as increased poverty and hunger (41). Reduced funding for housing in Inuvik and the Beaufort-Delta region of Northern Canada has resulted in mass housing shortages, with one in seven people living without adequate shelter leading to an observed increase in mental illness and addiction (45). Insufficient investments in housing among First Nation communities in Canada has also resulted in unacceptable rates of mould in housing, with 21% of homes in Haisla First Nation contaminated with visible mould (74); health risks associated with mould exposure disproportionately affect children and can lead to respiratory distress, immunosuppression, cognitive difficulties, fatigue, and asthma (43). In Chile, neoliberal policies have resulted in limited health care provision for Indigenous communities, with some communities experiencing neglect of health care facilities, for example, the Williche only had a health centre founded in 2007 (65). Although Aboriginal Community Controlled Health Services (ACCHS) in Australia are mandated to provide culturally responsive health services that reflect Aboriginal values and workforce, the neoliberalisation of care, which prioritises profit focused values and a non-Aboriginal workforce, has resulted in a lack of Aboriginal leadership for some ACCHS, *“Back then your driving force was your Aboriginal Health Worker. Your managers or funding bodies finalised it and you ran it. Now its driven by people who don’t know what is going on in the community. So how do they know what is best for us?”* (50). The influence of powerful groups on provision of dental service models in the United States exemplifies the inadequate investment in Indigenous oral health. For example, 36,000 residents of Oglala Lakota are being serviced by only nine dentists and the Navajo Nation has an average ratio of 32.3 dentists per 100,000 individuals (71). Reduced public expenditure has also seen an increase in *redistributive practices that harm Indigenous dignity and social wellbeing* in Canada (38). While promotion of Indigenous self-governance and self-determination might appear to benefit neoliberal ideologies of personal autonomy and privatisation, the reduction of public expenditure undermines Indigenous autonomy (38), “the redistributive tactics of neoliberalism are wide ranging, sophisticated, frequently masked by ideological gambits but devastating for the dignity and social well-being of vulnerable populations and territories” (75).

Reduced public expenditure has also led to an *increase in social risk* among Indigenous communities in Aotearoa/New Zealand (51, 52, 71), Australia (49, 71), Canada (44, 71), and the United States (57, 71). In reflecting on the impacts of neoliberal policies on Māori health, through reduced income, social fragmentation undermining social capital, and increased housing costs, Hodgetts refers to neoliberalism as “social experimentation” between “the haves” and “the have nots” (52). This notion is exemplified by the steady increase in Māori life expectancy observed through the 1950s to 1970s and the subsequent plateau in the 1980s, when social reforms were introduced, while Pakeha (white non-Maori New Zealanders) life expectancy continued to rise (52). This sentiment was echoed by Shorten, a member of parliament in Australia, who asserted in 2004 that costs of transitioning in the dynamic world economy were being borne too greatly by Aboriginal and Torres Strait Islander peoples, as evidenced by significantly lower life expectancies than non-Indigenous Australians (49). This risk-shifting was explained by Barnett and Bagshaw as a negative relationship between austerity and health where, *“those already disadvantaged bear the consequences of deterioration in the determinants of health”* (51). Jamieson and colleagues (70, 71) detail how neoliberalism’s contribution to wealth inequities disproportionately impacts Indigenous populations in Aotearoa/New Zealand, Australia, Canada, and the United States which increases social risk, manifested as lower income and under- and unemployment. Social risk also relates to job security and health insurance as detailed by an Alaskan Native fisherman, *"It’s tough, because you’re considered self-employed, so you get hit with higher taxes. Taxes are nuts. And you of course, have no health care, that’s a big part of that. It doesn’t offer a lot of stability, I think that’s why people think about settling down with someone and starting a family and it’s like, ‘well I don’t have insurance and don’t have a guaranteed job this month and what would happen?”* (57). The increase in social risk observed among Māori communities, and indeed globally, is not an accidental phenomenon but a direct result of neoliberal policies that impact mortality as well as morbidities, including obesity, mental health, and health risk behaviours (51).

#### Personal autonomy

Four overarching generative mechanisms related to the influence of personal autonomy on Indigenous health outcomes. *Failure to recognise and appreciate Indigenous knowledges and understandings of wellbeing* impacted the health and wellbeing of Indigenous communities in Canada (38), Mexico (66), Bolivia (64), and Aotearoa/New Zealand (54). Many Mayan communities are rooted in traditions that often clash with Western understandings of disease and healing; for example, some Mayan peoples employ a communitarian approach to illness where decisions are taken communally rather than autonomously and mental reasoning is not taken into consideration when making healthcare decisions, because the heart is believed to be the receptor of the divine essence which enables people to use their good sense. It is critical to build bridges of communication and trust to facilitate healthcare for Mayan communities, this was not possible during the crisis state of the Covid-19 pandemic, which unfortunately saw increasing vulnerability among Mayan communities (66). Failure to appreciate Indigenous knowledges risks child wellbeing in Bolivia, where the adaptive cultural resources for protecting health are dismissed in healthcare systems, despite the strong association between maternal knowledge and child health outcomes, as indicated by blood markers of immune function, height, and skin-fold thickness (64). The manifestation of intersecting forms of dispossession lead to spaces of deprivation and exclusion in health care settings for Namg First Nation in Canada, demonstrating how historic colonial relations transform to neo-colonial relations. Repossession of space for Indigenous health equity must come from the perspective that people, structures, practices, and policies within health care shape and create experiences of First Nation health (38). In conducting research regarding weight management among Māori communities, researchers discussed the neoliberal sanitisation of holistic Māori health views, wherein media and public health campaigns focus on individualisation. This has ultimately enabled the aggregation of Māori as a distinctive group, whose comparison to the rest of the population can then be folded back onto the Māori collective in a disciplinary manner (54).

The *association of worth or morality with health status* disempowers Māori individuals in Aotearoa/New Zealand (54) and enables neoliberal-ableism in Canada (44). Neoliberal biopolitics of the body in Aotearoa/New Zealand maintain the belief that not only is weight loss entirely reasonable, but that it is the only ethical position for an overweight or obese individual to inhabit. In Canada, concepts of neoliberal-ableism lead to a willingness of Indigenous peoples with a disability to sacrifice one’s self to death due to a perception of low productivity or high cost of dependency; in this space Indigenous peoples with a disability, “become window dressing[s] for the pervasive logic of neoliberal-ableism and sacrificial citizens” (44).

The neoliberal promotion of personal autonomy often *disregards socioeconomic inequities and historic injustices experienced by Indigenous peoples*. The neoliberal introduction and reinforcement of high living costs and poverty, globally, are not an isolated incident of social disadvantage but are compounded by historic trauma, colonial values, institutional racism, existing health, social, economic, and political disparities. For Inuit, Amazonian, and Alaskan Native communities, an existing prevalence of food insecurity increases sensitivity to nutritional deficiencies caused by disrupted traditional food systems (68). The focus on personal autonomy related to the medicalisation of consequences of Aboriginal and Torres Strait Islander health inequities relates to the expression of disadvantage, such as substance use, violence, and suicide, and forces a focus on the presumed mediators of these expressions and encourages service solutions (47). Service solutions have limited indications of improvement in outcomes and disregard the complexity of socioeconomic and historic injustices experienced by Indigenous peoples (47). For American Indian and Alaskan Native communities in the United States, there is an expectation that a relationship of trust with the United States government will improve relationships with communities through the provision of health, social services, and government. This expectation dismisses the forced assimilation into mainstream America that removed American Indian and Alaskan Native communities from their traditional lands and customs and has resulted in sustained poverty, trauma, and health inequities such as chronic disease, substance use, poor mental health, and mortality compared to non-Indigenous Americans (71).

Finally, the neoliberal ideology promotion of personal autonomy enables *blaming of poor health outcomes on “Indigenous lifestyles”* which results in classist social derision of purchasing decisions, lifestyles, and subsequent health outcomes (70); examples from Canada (42), Venezuela (62), and Australia (48, 50) illustrate the impact of this generative mechanism on Indigenous health. Remote Aboriginal and Torres Strait Islander communities in Australia have been seen as ‘ungovernable’ spaces that are distant, both metaphorically and physically, from the ‘civilised’ world; the assumption that these communities are ‘failures’ with an ‘enclave of social problems,’ limits the pursuit of health equity and permits the continuation of poor health (48). Perspectives among ACCHS clinicians in Australia is torn between neoliberal ideologies of personal autonomy and Indigenous cultural systems which preserve social protection; the paradox of blaming appointment attendance on client ‘lifestyle’ in a community controlled service is revealing, *“I think some of them are just slack to make the appointments, you know. They know all about it, but they’re slack. It’s low down on the list of priorities. They just see it as a big disruption to their, you know, lifestyle”* (50). In Canada, neoliberal public health messaging emphasises women’s responsibilities for healthy pregnancies and babies highlighting the need to avoid alcohol, tobacco, and other drugs. The responsibility to provide maternal education campaigns targeting foetal alcohol syndrome have been shifted to Indigenous communities due to the belief that births affected by foetal alcohol syndrome are ‘entirely preventable’ and an unjustifiable ‘cost to communities’ (42). In Venezuela, the manipulation of the cause of cholera, including unhygienic and unsanitary conditions, was utilised by the government to conveniently blame the spread of disease on Warao ‘Indigenous lifestyle and conditions’ (62).

#### Deregulation that facilitates economic activity

Two overarching generative mechanisms related to the influence of deregulation on Indigenous health outcomes. The *inequitable provision of healthcare on the basis of race* was discussed among Indigenous communities in Venezuela (62), Australia (48), Brazil (60), and the United States (56). The implications of budget cuts for Indigenous communities in the United States was evident early in the neoliberal era; in 1983, Walker critiqued a Reagan Administration proposal to define the term “Indian” by the amount of “Indian blood” an individual possessed as another means to reduce the number of people eligible for federally funded health care, *“excluding Indian people from [Indian Health Service] funding will neither solve human nor budgetary problems. The likely result will be more Indian deaths and hospital emergency visits. The cuts proposed in urban Indian health services are but another example of an inconsistent Federal policy that chooses to recognize Indian people when convenient for the government and to ignore them when the consequences of Federal action result in human suffering”* (56). Since this time, neoliberal policies have continued to directly influence systemic racism through the promotion of competition and support for groups in power; systemic racism originates in the operation of societal forces which are increasingly neoliberal. The impact of racism on Indigenous oral health inequities have been empirically examined in Canada, Australia, Aotearoa/New Zealand, and the United States (71). In Venezuela, the death of Warao community members during a cholera outbreak has been attributed to the disease-favouring conditions created by the impact of racism on the provision of health care, water, and waste treatment by government (62). In Australia, Shared Responsibility Agreements were used in place of health care provision for some Aboriginal and Torres Strait Islander communities. For example, the 2004 Mulan agreement specified that community would, *“make sure kids shower everyday, wash face[s] twice a day… ensure that rubbish bins are at every house and that they are emptied twice each week… ensure that household pest control happens four times each year… [and] ensure that petrol sold through the store is not used for petrol sniffing.”* In return, this community would receive Australian government contributions towards the provision and installation of fuel bowsers, with compliance tested by state government regularly via skin and worm infection assessments of children (48). In Brazil, Indigenous peoples suffered disproportionately from Covid-19 due to restricted access to an already precarious health system fraught with racist, structural neglect that has been likened to “policies of extermination” by Indigenous movements. Limited healthcare accessibility was compounded by government attempts to continue neoliberal development, undermining Indigenous and environmental rights, during what the Minister of Environment in Brazil deemed *“a moment of calm while the press is focusing on the pandemic”* (60). Mining of Indigenous territories was deemed an ‘essential service’ in Brazil and ultimately introduced Covid-19 to nearby Indigenous communities with limited health care infrastructure (60).

Neoliberal ideologies of deregulation also *perpetuate colonial and patriarchal structures* through power structures, particularly targeting Indigenous and female rights. Pictou asserts that one of the greatest tragedies of Canada’s neoliberal approach is its exclusionary practices that limit Indigenous women’s ability to hold leadership roles and partake in official negotiation processes (41). Women are traditionally Land and Water protectors in many First Nations communities; their continued struggle against neoliberal and colonial gender discrimination disrupts traditional duties and limits their ability to protect traditional lands and communities (41). Patriarchal structures are also upheld by neoliberal ideologies in Peru, where Indigenous women have been instrumentalised as citizens and called upon to regulate their ‘overly fecund bodies’ to drive the country’s desired poverty reduction; due to their exclusion from full rights, many Indigenous peoples were forced, rather than asked, to limit their fertility through sterilisation (67).

### Generative mechanisms of indigenous resistance

#### Competitive and private markets

Indigenous communities in the United States (55), Brazil (60), and Guatemala (59) have demonstrated strong *community resistance to industrial development*. Contexts of neoliberal government negligence in Brazil has seen emergence of grassroots initiatives and resistance. The Mebengokrê-Mekranoti blocked the highway utilised for transportation of goods, demanding government support against deforestation and their fight against Covid-19; despite the lack of support and the use of violence against the blockade, they continued to stand firm (60). Indigenous communities in the northeast of Brazil recognised their invisibility to the state and took autonomous actions to prevent the spread of Covid-19 in their communities, *“We had to compensate for the absence of the state, they didn’t have a platform of action. The blockades are one action within reach of Indigenous peoples to help mitigate the impacts”* (60, 76). The Mayan resistance movement, ‘Peaceful Resistance La Puya’ in Guatemala has defended their local water and health from mining development for a decade through the maintenance of a 24-h encampment; this group has also been successful in anti-corruption cases, with many government members now incarcerated (59). In the United States, Indigenous environmental activists similarly fight to protect the health of their community members, *“Well, in my community we had an oil-field waste site… I was living in a toxic town… still do… We got together, kids at the bus stop and parents would say their kids were sick with asthma and every month it’s the same thing. And then we realized they had dumped toxic waste inside of our community. So I became an environmentalist, not by choice but… we fought for seven years against the oil and gas company… I was fighting for the rights of my community and my kids to live a normal, happy life as we knew it in a small town… we watched kids with no asthma become asthmatic. We watched kids would grow up with… severe diarrhea, nausea, dizziness, vertigo. It’s different effects from all these chemicals, from the oil and gas industry”* (55).

#### Reduced public expenditure on infrastructure

In response to the rise in neoliberalism, the Māori health movement in Aotearoa/New Zealand has *advocated for increased investment in Indigenous health*. Led by Smith, an influx of Indigenous scholars and allies forced a reversal of a national funding agency decision to withdraw funding for Māori research centres, enabling the continuation of Indigenous-led health research in Aotearoa/New Zealand (53). This advocacy validated the importance of Māori health research by Māori researchers and for Māori communities; funding set aside by the Health Research Council of New Zealand was specifically dedicated to community-based research, research development, post-graduate scholarships, and studentships for Māori research and researchers (54).

#### Community autonomy

Indigenous communities in Brazil (60, 61), Canada (41), and Australia (50) have *utilised traditional practices to reduce reliance on neoliberal systems*. Food sovereignty and subsistence farming in Brazil has helped communities avoid Covid-19 exposure (60) and reduced reliance on industrialised products (61). Subsistence farming practiced by the Guarani is a conscious strategy of cultural reproduction and neoliberal resistance that allows the community to maintain sovereignty and independence from industrialised products, *“As you see the cassava there, everyone has planted. Corn also, there is plenty of corn, and it prevents us from buying food outside”* (61). Similarly, Indigenous women in Canada have demonstrated profound resilience against neoliberal forces, teaching alternative ways to live in harmony with each other and with the natural ecosystems that sustain them, leading land-based, water-based, and food practices (41). The continued provision of culturally responsive health care by ACCHS and strong Aboriginal workforces in Australia ensures continuity of care and positive community engagement, *“The good thing is we sort of know everyone in the community so we can go to their house and if they are not there then we can ask if they know where such and such are and if they are not there then they are at this house. We will do this until we can find them”* (50).

#### Deregulation that facilitates economic activity

*Ally advocacy for the protection of Indigenous rights* was discussed both in the Canadian (38) and Brazilian (60) contexts. Nurses in Canada have resisted the ‘naturalisation’ of language decline and dispossession experienced by Indigenous peoples by orienting actions toward opportunities of repossession, engaging with Indigenous peoples and acknowledging how dispossession creates disconnection and oppression which impacts health care inequities (38). Despite mandates to reduce support activities for Indigenous health during the Covid-19 pandemic, allies in the Brazilian Senate approved an ‘Emergency Plan to Combat Coronavirus in Indigenous Peoples.’ Although this bill was initially vetoed by Bolsonaro and hundreds of Indigenous peoples died due to Covid-19, Congress eventually overruled Bolsonaro’s decision and instated support for communities facing a public health crisis (60).

## Discussion

Due to the vast health inequities experienced by Indigenous peoples around the world and the pervasive nature of neoliberal ideologies in health discourse, service delivery, and experiences of health inequities, this systematic scoping review aimed to identify the generative mechanisms through which neoliberalism impacts Indigenous health, globally. Utilising principles of qualitative systematic review meta-aggregation methodologies (35), 100 pieces of evidence from 38 included articles were synthesised into 16 generative mechanisms and four generative mechanisms of resistance. Generative mechanisms were mapped against four core principles of neoliberalism: competitive and private markets, reduced public expenditure, personal autonomy, and deregulation that facilitates economic activity.

Indigenous communities have been subjected to social and environment destruction, greed, and militarisation for centuries (6, 8, 30); neoliberalism follows this pattern of oppression. As such, “the practical critique of neoliberalism embodied in Indigenous peoples’ resistance into the global market is one informed by an acute recognition of not only the global dimensions of such resistance but also an acknowledgement of anti-imperialist struggles stretching back over many hundreds of years” (77). The evidence synthesised in this review further substantiates the argument that neoliberalism perpetuates colonial ideologies that continue to oppress and marginalise Indigenous peoples across the world. Neoliberalism inhibits Indigenous peoples’ right to self-determination, which was a critical component to the historical attempts to assimilate Indigenous peoples in colonised countries (21). Raghavan coined the phrase “re-colonisation” in 1990 (78) in relation to the extensive impacts of neoliberalism on Indigenous peoples. Bagh expanded on this idea noting, “re-colonization is the embedding and re-embedding of neoliberalism utilizing multiple avenues including institutional, state, corporate and intellectual pressure” (79). The multiple levels across which the generative mechanisms identified in this review exist provides further evidence on how neoliberalism explicitly acts to embed colonial values across individual, community, state, national, and international contexts. In Smith’s (30) seminal decolonising methodologies work, she highlighted the colonial power frameworks that act to erase and subordinate Indigenous knowledges and worldviews. Similar processes of neoliberal subalternation of Indigenous knowledges regarding understandings of health and healing was highlighted in this review wherein, health services fail to incorporate Indigenous values and health providers fail to incorporate Indigenous knowledges. While public health researchers have gone some way to acknowledge, incorporate and work to intervene on the impacts of social determinants of health (80), largely missing from this dominant narrative is genuine acknowledgment of the ways in which neoliberal states and societies continue to deny Indigenous peoples’ rights to self-determination and the ways in which we, as actors within neoliberal systems, reproduce colonial power relations (21).

Due to the embedded subalternation of Indigenous knowledges within dominant colonial discourses, embracing Indigenous knowledges and decolonising frameworks in governance, research, and policy is necessary to critically refuting neoliberal structures and ideologies. Failing to value Indigenous analyses of neoliberalism and Indigenous strategies of resistance is to seriously restrict meaningful and transformative action for dominant health discourse and social, political, and economic order (21). Sovereignty and self-determination for Indigenous peoples needs to be the bottom line for a different world order that mandates health equity (21). For example, the Sámi peoples in Norway are constitutionally recognised and have their own governance system that recognises the rights of Sámi children to speak and receive education in their traditional languages. The health inequities among Sámi peoples in Norway are of a lesser magnitude than the profound inequities experienced between Indigenous and non-Indigenous peoples in other parts of the world (81). The Lowitja Institute in Australia is an Aboriginal and Torres Strait Islander community-controlled national health research institute leading the way in research sovereignty among communities. The Lowitja Institute is dedicated to the strength and agency of Indigenous communities and researchers and prioritises investment in community driven health research, mobilisation of research knowledge, and enhancement of the Indigenous research workforce capability, and sustainability (82). Similarly, the Aboriginal Community Controlled Health sector in Australia ensures community ownership of health, has strengthened health outcomes, and has been recognised as a best practice example for the implementation of the right to self-determination highlighted in the United Nations Declaration on the Rights of Indigenous Peoples (83). These examples of governance models related to Indigenous health and research directly resist neoliberal ideologies and honour Indigenous knowledges and epistemologies.

Largely, recommendations and outcomes from studies included in this review called for a change in discourse and epistemologies regarding neoliberal ideologies. Suggestion on how to change neoliberal models and societal discourses include the development of social democratic governance models with ethical political ideologies that respect Indigenous self-determination and prioritise Indigenous leadership (5, 15). As academics, policy makers and researchers, we have a choice to either act to resist neoliberal frameworks and promote decolonial values or to be complicit in the maintenance of neoliberalism and colonialism. We contend that all actors in neoliberal societies are ethically compelled to lead work, and lives more generally, that pushes society into a just and equitable future by challenging neoliberal models that continue to harm Indigenous health outcomes, as evidenced by the findings of this review. As suggested by Borde and Hernández (5), we must move beyond the social determinants of health framework to clarify the “causes of the ‘causes of the causes,’” failing to do so reproduces processes that sustain health inequities (5, 80). The expectation that health inequities created and sustained by neoliberal systems and colonial values can be solved while maintaining these very structures is idealistic and completely unrealistic. Belief in ‘win–win’ solutions to health inequities embedded in neoliberal structures remain superficial and reliant on an idea of civil identity and autonomy that fundamentally supports colonial values. In the absence of comprehensive analysis of power, global politics, society, and societal relations, social determinants of health are inadequate indicators of health inequities (5). Failure by non-Indigenous people to end the silence of neoliberalism’s impacts on health is problematic and demonstrates the selective and colonial nature through which ‘social justice’ operates (21, 84).

We maintain that all considerations of social and health inequities, particularly those related to Indigenous wellbeing, must not only consider the innate relationship between neoliberal political economies and health outcomes in research development, design, implementation, and analysis, but must explicitly investigate the impact of neoliberalism on the reported health inequity. Investigations must move beyond the inevitability of colonial, neoliberal, deficit health discourse to develop a more nuanced understanding of the generative mechanisms through which neoliberalism amplifies health inequities (19, 20, 70, 85), identifying the structural and systemic perpetrators of Indigenous health inequity. In doing so, Indigenous leadership and perspectives must be privileged and amplified to ensure a critically robust analysis that challenges the acceptance and reproduction of neoliberal impacts on both Indigenous and non-Indigenous wellbeing (86).

### Strengths and limitations

This review considered the impact of neoliberal generative mechanisms in relation to an Indigenous-defined understanding of wellbeing, which included connections to traditional lands (33). Generative mechanisms of resistance were an incidental finding of this work, future work must centre stories of Indigenous strength and resistance to neoliberalism to document the widespread success of global Indigenous communities, share knowledges related to these successes, and inspire more work in this space. While the authors made all attempts to limit publication bias through the inclusion of all languages and locations across the world, as well as grey literature, limitations remain. Systematic searches fail to capture knowledges and stories not contained within written and published literature. Further, terminology around neoliberalism can vary and 21 studies included in this review did not explicitly define neoliberalism in relation to their works. Studies that did not specify the impact of ‘neoliberalism’ and instead used terminologies such as bureaucratization or free market in their work were excluded to maintain boundaries of a systematic and rigorous methodology. As such, it is likely that this work captures only a snapshot of the ways in which the pervasive nature of neoliberal ideologies impacts Indigenous wellbeing.

## Conclusion

Actors within neoliberal societies must resist dominant epistemological, ontological, and praxiological stances that reinforce the supremacy of colonial values and subalternation of Indigenous ways of knowing, being, and doing to begin effectively addressing Indigenous health inequities. In line with the evidence generated by this review, we recommend the following: (1) consideration and investigation of neoliberal ideologies and structures as common practice in health equity scholarship; (2) explicitly attributing circumstances of inequitable health to neoliberalism, where evidenced, and holding neoliberal actors accountable for the consequences of their decisions; and most importantly, (3) designing research and policies in ways that honour and amplify Indigenous resistance to neoliberalism and assertions of self-determination.

## Supplementary Information

**Additional file 1.** Preferred Reporting Items for Systematic reviews and Meta-Analyses extension for Scoping Reviews (PRISMA-ScR) Checklist.

**Additional file 2.** Search Strategy.

**Additional file 3.**

**Additional file 4.** Generative mechanisms.

## Data Availability

The data supporting the conclusions of this article are available in the published literature included in this review.
