# Supplementary material for: The impact of neoliberal generative mechanisms on Indigenous health: a critical realist scoping review
Source: Global Health. 2022 Jun 15;18:61. doi: 10.1186/s12992-022-00852-2 (PMC9199313; doi:10.1186/s12992-022-00852-2)
Supplement: Supplementary file 2 — Additional file 2. Search Strategy. [file 12992_2022_852_MOESM2_ESM.docx]

Supplemental X – Search Strategy

**PubMed Logic Grid**

| Neoliberalism | Indigenous Population | Health |
| --- | --- | --- |
| neoliberal*[tw] OR “corporate determinants of health”[tw] OR “commercial determinants of health”[tw] OR globalisation[tw] OR bureaucratization[tw] OR commodification[tw] OR “Economic competition”[mh] OR privatization[mh] OR “health care economics and organizations”[mh] | "first nation*"[tw] OR "pacific islander*"[tw] OR "torres strait islander*"[tw] OR aborigin*[tw] OR alaska*[tw] OR aleut*[tw] OR amerind*[tw] OR arctic[tw] OR aymara[tw] OR bushmen[tw] OR chukchi[tw] OR chukotka*[tw] OR circumpolar[tw] OR eskimo*[tw] OR greenland*[tw] OR hmong[tw] OR indian*[tw] OR indigen*[tw] OR inuit*[tw] OR inupiaq[tw] OR inupiat[tw] OR khanty[tw] OR maori*[tw] OR mapuche[tw] OR metis[tw] OR native*[tw] OR navaho*[tw] OR navajo*[tw] OR nenets[tw] OR quechua[tw] OR sami[tw] OR samoan*[tw] OR siberia*[tw] OR skold[tw] OR tribal[tw] OR tribe*[tw] OR xingu*[tw] OR yup’ik[tw] OR yupik[tw] OR zuni[tw] OR "African continental ancestry group"[mh] OR "African continental ancestry group"[mh] OR "Asian continental ancestry group"[mh] OR "Oceanic ancestry group"[mh] OR "arctic regions"[mh] OR "ethnic groups"[mesh] | “health”[mh] OR “health inequity”[tw] OR “health equity”[tw] OR “health equity”[mh] |

**EMBASE Logic Grid**

| Neoliberalism | Indigenous Population | Health |
| --- | --- | --- |
| 'commodification'/exp OR 'economic aspect'/exp OR 'globalisation'/exp OR 'health economics'/exp OR neoliberal*:ti,ab,kw OR 'corporate determinants of health':ti,ab,kw OR 'commercial determinants of health':ti,ab,kw OR globalisation:ti,ab,kw OR commodification:ti,ab,kw OR bureaucratization:ti,ab,kw OR privatization:ti,ab,kw | 'indigenous population':ti,ab,kw OR 'indigenous people':ti,ab,kw OR 'Samoan (people)':ti,ab,kw OR 'First Nation':ti,ab,kw OR 'Pacific Islander':ti,ab,kw OR 'Torres Strait Islander':ti,ab,kw OR 'Black person':ti,ab,kw OR 'Alaska Native':ti,ab,kw OR 'Aleut (people)':ti,ab,kw OR 'Amerind people':ti,ab,kw OR 'Aymara (people)':ti,ab,kw OR 'Chukchi (people)':ti,ab,kw OR 'Chukotka':ti,ab,kw OR 'Eskimo-Aleut people':ti,kw,ab OR 'Greenland':ti,ab,kw OR 'Hmong (people)':ti,kw,ab OR 'Indian':ti,ab,kw OR 'indigenous people':ti,kw,ab OR 'Inuit':ti,kw,ab OR 'Inupiat (people)':ti,kw,ab OR 'Khanty (people)':ti,ab,kw OR 'Maori (people)':ti,kw,ab OR 'Mapuche (people)':ti,kw,ab OR 'indigenous people':ti,kw,ab OR 'Navajo (people)':ti,kw,ab OR 'Nenets (people)':ti,kw,ab OR 'Quechua (people)':ti,kw,ab OR 'Sami (people)':ti,kw,ab OR 'Yupik (people)':ti,ab,kw OR 'Zuni (people)':ti,ab,kw OR 'Asian continental ancestry group':ti,ab,kw OR 'Oceanic ancestry group':ti,ab,kw OR 'Arctic':ti,ab,kw OR 'ethnic group':ti,kw,ab | 'health'/exp OR 'health equity'/exp OR 'health inequity'/exp OR 'health equity':ti,ab,kw OR 'health inequity':ti,ab,kw |

**Scopus Logic Grid**

| Neoliberalism | Indigenous Populations | Health |
| --- | --- | --- |
| (TITLE-ABS-KEY (Neoliberal* OR “corporate determinants of health” OR “commercial determinants of health” OR globalisation OR bureaucratization OR commodification OR “health economics” OR privatization) | TITLE-ABS-KEY ("first nation" OR "first nations" OR "pacific islander" OR "pacific islanders" OR "torres strait islander" OR "torres strait islanders" OR aborigin* OR alaska* OR aleut* OR amerind* OR arctic OR aymara OR bushmen OR chukchi OR chukotka* OR circumpolar OR eskimo* OR greenland* OR hmong OR indian* OR indigen* OR inuit* OR inupiaq OR inupiat OR khanty OR maori* OR mapuche OR metis OR native* OR navaho* OR navajo* OR nenets OR quechua OR sami OR sami OR samoan* OR siberia* OR skold OR tribal OR tribe* OR xingu OR yup'ik OR yupik OR zuni OR "African continental ancestry group" OR "African continental ancestry group" OR "Asian continental ancestry group" OR "Oceanic ancestry group" OR "arctic region") | (TITLE-ABS-KEY (health OR health equity OR health inequity) |

**Web of Science Logic Grid**

| Neoliberalism | Indigenous Populations | Health |
| --- | --- | --- |
| TI=(neoliberal* OR “corporate determinants of health” OR “commercial determinants of health” OR globalisation OR bureaucratization OR commodification OR “health economics” OR privatization) OR AB=(neoliberal* OR “corporate determinants of health” OR “commercial determinants of health” OR globalisation OR bureaucratization OR commodification OR “health economics” OR privatization) OR AK=( neoliberal* OR “corporate determinants of health” OR “commercial determinants of health” OR globalisation OR bureaucratization OR commodification OR “health economics” OR privatization) | TI=(first nation* OR pacific islander* OR torres strait islander* OR aborigin* OR africa* OR alaska* OR aleut* OR amerind* OR arctic OR aymara OR bushmen OR chukchi OR chukotka* OR circumpolar OR eskimo* OR greenland* OR hmong OR indian* OR indigen* OR inuit* OR inupiaq OR inupiat OR khanty OR maori* OR mapuche OR metis OR native* OR navaho* OR navajo* OR nenets OR quechua OR sami OR samoan* OR siberia* OR skold OR tribal OR tribe* OR xingu* OR yup’ik OR yupik OR zuni OR African continental ancestry group OR African continental ancestry group OR Asian continental ancestry group OR Oceanic ancestry group OR arctic regions OR ethnic groups) OR AB=( first nation* OR pacific islander* OR torres strait islander* OR aborigin* OR africa* OR alaska* OR aleut* OR amerind* OR arctic OR aymara OR bushmen OR chukchi OR chukotka* OR circumpolar OR eskimo* OR greenland* OR hmong OR indian* OR indigen* OR inuit* OR inupiaq OR inupiat OR khanty OR maori* OR mapuche OR metis OR native* OR navaho* OR navajo* OR nenets OR quechua OR sami OR samoan* OR siberia* OR skold OR tribal OR tribe* OR xingu* OR yup’ik OR yupik OR zuni OR African continental ancestry group OR African continental ancestry group OR Asian continental ancestry group OR Oceanic ancestry group OR arctic regions OR ethnic groups) OR AK= (first nation* OR pacific islander* OR torres strait islander* OR aborigin* OR africa* OR alaska* OR aleut* OR amerind* OR arctic OR aymara OR bushmen OR chukchi OR chukotka* OR circumpolar OR eskimo* OR greenland* OR hmong OR indian* OR indigen* OR inuit* OR inupiaq OR inupiat OR khanty OR maori* OR mapuche OR metis OR native* OR navaho* OR navajo* OR nenets OR quechua OR sami OR samoan* OR siberia* OR skold OR tribal OR tribe* OR xingu* OR yup’ik OR yupik OR zuni OR African continental ancestry group OR African continental ancestry group OR Asian continental ancestry group OR Oceanic ancestry group OR arctic regions OR ethnic groups) | TI=(health OR health equity OR health inequity) OR AB=(health OR health equity OR health inequity) OR AK=(health OR health equity OR health inequity) |

**ProQuest Central Logic Grid**

| Neoliberalism | Indigenous Populations | Health |
| --- | --- | --- |
| noft(Neoliberal* OR “corporate determinants of health” OR “commercial determinants of health” OR globalisation OR bureaucratization OR commodification OR “health economics” OR privatization) | noft("first nation*" OR "pacific islander*" OR "torres strait islander*" OR aborigin* OR alaska* OR aleut* OR amerind* OR arctic OR aymara OR bushmen OR chukchi OR chukotka* OR circumpolar OR eskimo* OR greenland* OR hmong OR indian* OR indigen* OR inuit* OR inupiaq OR inupiat OR khanty OR maori* OR mapuche OR metis OR native* OR navaho* OR navajo* OR nenets OR quechua OR sami OR samoan* OR siberia* OR skold OR tribal OR tribe* OR xingu OR yup'ik OR yupik OR zuni OR "African continental ancestry group" OR "African continental ancestry group" OR "Asian continental ancestry group" OR "Oceanic ancestry group" OR "arctic regions") | noft(health OR health equity OR health inequity) |
