# Supplementary material for: The impact of neoliberal generative mechanisms on Indigenous health: a critical realist scoping review
Source: Global Health. 2022 Jun 15;18:61. doi: 10.1186/s12992-022-00852-2 (PMC9199313; doi:10.1186/s12992-022-00852-2)
Supplement: Supplementary file 3 — Additional file 3. [file 12992_2022_852_MOESM3_ESM.docx]

| **Author** | **Title** | **Year** | **Country** | **Indigenous community** | **Study Design** | **Aim** | **Main Outcome/Results** |
| --- | --- | --- | --- | --- | --- | --- | --- |
| Barges | Culture, territories, and confidence in food: An Anthropological view on health in the context of environmental pollution and socio-political tension | 2008 | Canada | Ojibway and Cree First Nations | Review | To show the physical, cultural, and socio-political impacts of the presence and exposure to pollutants in the environment. | Water pollution had an impact on people's minds, practices, science, and politics. It has contributed to Pan-Canadian and international awareness of a global and permanent problem. |
| Barnett & Bagshaw | Neoliberalism: what it is, how it affects health and what to do about it | 2020 | Aotearoa/New Zealand | Māori communities | Commentary | To discusses the consequences of neoliberalism on health. | To reverse the impacts of neoliberalism change is needed with: (i) the objective of equity of health outcomes; (ii) re-investment in health and social security; (iii) the rejection of the marginalisation of health professionals in decision-making, and (iv) a move to a more integrated health system. |
| Briggs | Stories in the time of cholera: Race and public health in Venezuela | 2002 | Venezuela | Warao communities | Commentary | To describe the blame and shunning of Warao communities during Cholera (1992). | There is a need to find ways to deal with disease and inequality that avoid blaming others, a paralysing defensive reaction that precludes a deeper search for answers that could avert a dumbfounding return to disease patterns that resemble those of the nineteenth century. |
| Browne & Stout | Moving Towards Nahi: Addressing Health Equity in Research Involving Indigenous People | 2012 | Canada | First Nations, Inuit, and Métis communities | Editorial | To reflect on the extent to which health disciplines shape the landscape of health research and to identify strategies for moving forward in partnership with Indigenous people. | Given the increasing adoption of culture as the panacea for improving their health, Indigenous people run the risk of being reduced to cultural beings for whom health interventions need not be more than one-dimensional. Transformational change for equity in relation to health, social conditions, and health care is needed. |
| Brown et al. | Our Land, Our Language: Connecting Dispossession and Health Equity in an Indigenous Context | 2012 | Canada | Namg First Nation | Qualitative exploratory study | To identify the implications for nurses when they strive for health equity and partner with First Nations communities. | If dimensions of the “social” are constituted through cultural identity and connections to the land, then the scope of nursing action directed towards increasing access to the social determinants of health will necessarily expand. By working with community members and cultural leaders, nurses can partner to create the spaces of repossession that are fundamental to experiences and contexts for achieving health and healthcare equity. |
| Dinorah Martinez et al. | “Cancer is in style”: lifestyle change and the perceived impact of globalization on Andean Indigenous communities in Ecuador | 2021 | Ecuador | Kichwa communities | Qualitative exploratory study (using focus groups) | To describe the perceptions of cancer among a Kichwa community. | Anthropological perspectives that conceptualize health issues like cancer within their historical, political, and social context are needed as health and illness are not just the product of access to health care but are rooted in the economic, political, and cultural dimensions of society. |
| Donders & Barriocanal | The influence of Markets on the Nutrition Transition of Hunter-Gatherers: Lessons from the Western Amazon | 2020 | Ecuador & Bolivia | Tsimane communities (Bolivia) and Huaorani communities (Ecuador) | Review | To review the market involvement of Indigenous communities in the Western Amazon and its consequences on diet, health, and well-being. | A better understanding of the complex relations between markets, diet, and health is needed for policy makers who aim to introduce changes that will impact traditional territories and equip Indigenous peoples with information to shape their decision-making processes to protect their health. |
| Duran | Managing Nutrition and Health in a Changing Climate: A Yellowknives Dene First Nation Perspective | 2015 | Canada | Dene First Nation | Dissertation | To develop Midrange theory that describes the management of nutrition and health within the context of climate change and global environmental change. | The Global Environmental Change-YKDFN model and findings enhanced localized dialogue regarding nutrition and ecosystem services among decision-makers that include Indigenous, federal, and territorial governments. |
| Durey et al. | The mouth as a site of structural inequalities; the experience of Aboriginal Australians | 2016 | Australia | Aboriginal and Torres Strait Islander communities | Review | To address the mouth as a site of structural inequalities through Aboriginal and Torres Strait Islander experiences. | There is a need to shift focus away from blaming Aboriginal people for their health choice and better contextualise oral health inequalities. Emphasis on non-judgmental, non-discriminatory, and respectful policy that takes into consideration lived experiences. |
| Ford | Indigenous Health and Climate Change | 2012 | Global | Global Indigenous communities | Review | To review research on Indigenous health and climate change and capture place-based dimensions of vulnerability. | The health impacts of change is significant and for some Indigenous populations the current risks pose inevitable loss of homeland, livelihoods, and culture. |
| Hanrahan | Water (in)security in Canada: national identity and the exclusion of Indigenous peoples | 2017 | Canada | Nunatsiavut Inuit | Review | To demonstrate how neoliberalism legitimises decentralised water governance in Canada, which promotes and maintains environmental inequality. | Genuine progress requires a move away from Canada’s long-standing commitment to market principles as paramount and the concurrent notion that water must be earned by communities, rather than treated as the right it is in Canadian and international law. |
| Hodgetts et al. | Media Coverage of ‘Decades of Disparity’ in Ethnic Mortality in Aotearoa | 2004 | Aotearoa/New Zealand | Māori | Review | To explore the media coverage of the report ‘Decades of Disparity’, which proposed that neoliberal policies have negatively impacted mortality rates for Maori and Pacific peoples. | Coverage of the disparities report illustrates the benefits of public health researchers engaging in symbolic interventions. The analysis constitutes an effort to learn how we might improve such activities, while fostering the development of a better understanding of how social power relations and vested interests in society frame disparities in health. |
| Hovelsrud et al. | Arctic Societies, Cultures, and Peoples in a Changing Cryosphere | 2011 | Arctic | Arctic Indigenous communities | Review | To review how changes in sea ice, snow cover, lake and river ice, and permafrost will affect economy, infrastructure, health, livelihoods, culture, and identity. | In addition to the direct effects of a changing cryosphere, human society is affected by indirect factors, including industrial developments, globalization, and societal changes, which contribute to shaping vulnerability and adaptation options. |
| Hunter | Indigenous mental health: the limits of medicalised solutions | 2020 | Australia | Aboriginal and Torres Strait Islander communities | Commentary | To review the social and political factors contributing to the intransigence of vulnerability considering neoliberal political change. | While addressing underlying social vulnerabilities lies beyond clinical practice, the health sector has privileged access to and capacities to influence the critical neurodevelopmental period during which transgenerational vulnerability is informed and social disadvantage entrenched. Bipartisan political commitment and investment in Indigenous agency is needed. |
| Jamieson et al. | How Neoliberalism Shapes Indigenous Oral Health Inequalities Globally: Examples from Five Countries | 2020 | United States, Canada, Australia, Aotearoa, Norway | Indigenous communities from the 5 included countries | Commentary | To provides insights to how neoliberalism shapes oral health inequalities among Indigenous societies at a global level. | We posit that all socially marginalised groups are disadvantaged under neoliberalism agendas, but that this is amplified among Indigenous groups because of ongoing legacies of colonialism, institutional racism and intergenerational trauma. |
| Jamieson et al. | Neoliberalism and Indigenous oral health inequalities: a global perspective | 2021 | Global | Global Indigenous communities | Commentary | To structure a framework that encapsulates neoliberalism in the context of global oral health, through a social justice and human rights lens. | Neoliberalism impacts Indigenous oral health globally through policies structuring social resources and insidious psychosocial processes and constructs of shame. Reducing inequalities in Indigenous oral health at a global level requires robust policy recommendations and social change within our current socio-political context. |
| Lawrence & Gibson | Obliging Indigenous Citizens: Shared responsibility agreements in Australian Aboriginal communities | 2007 | Australia | Aboriginal and Torres Strait Islander communities | Case Study | To understand how the ethical conduct of Indigenous people is being debated, conceptualised, and executed by both Government and Indigenous people themselves. | Seemingly trivial practices of hygiene are linked to health, to social services, and to the ethical being of Indigenous people. This construction of citizenship works through a hierarchical scale of capacities and a view of culture in which Indigenous people are invariably deemed to be deficient in liberal virtues. |
| Liddell & Kingston | “Something Was Attacking Them and Their Reproductive Organs”: Environmental Reproductive Justice in an Indigenous Tribe in the United States Gulf Coast | 2021 | United States | Indigenous community in the Gulf Coast | Qualitative descriptive study (Community-based participatory action) | To apply the environmental reproductive justice frame- work to the reproductive health experiences described by women who self-identify as Indigenous. | Findings of this study reveal that central components of reproductive justice, including the ability to have children and the ability to raise children in safe and healthy environments, are undermined by environmental justice issues in the community, including high rates of chronic healthcare issues and infertility. |
| Lucas | Dynamics of food consumption in a Q'eqchi' Maya community | 2017 | Guatemala | Q’eqchi’ Maya community | Dissertation | To examine how nutrition transitions effect identity, locality, and economy in a Q’eqchi’ Maya aldea. | Fortified processed foods, branding, food and identity, globalization, the presence of old food associations, the impact of new food associations, and impact social institutions and prevent Q’eqchi’ Maya from meeting basic needs. |
| Menton et al. | The COVID-19 pandemic intensified resource conflicts and indigenous resistance in Brazil | 2021 | Brazil | Indigenous peoples in Brazil (various communities) | Mixed Methods (Interviews & Social Media audits) | To understand how Indigenous peoples have responded to the spread of COVID-19 in the face of active environmental conflicts. | At the national level, Indigenous movements, were able to draw on social media and Indigenous led court cases to help counteract the ‘genocide by omission’ that has been worsened by the pandemic. |
| Moore & Moore | Guatemala's land defenders are still their own best defence | 2021 | Guatemala | Mayan Indigenous communities | News article | To explain the impacts of large-scale extraction of gold, silver, and other metals | Indigenous resistance will continue despite efforts to destabilize Guatemalan government and legal systems. |
| Pictou | Food, Land and Treaty | 2017 | Canada | Mi'kmaq First Nation communities | Commentary | To share stories about the struggles of Indigenous women today in Mi'kmaki and across Canada against neoliberal extensions of colonialism, racism, and sexism. | All food policies must include Indigenous women in their development and implementation to ensure Indigenous food sovereignty. |
| Prussing & Newbury | Neoliberalism and Indigenous knowledge: Māori health research and the cultural politics of New Zealand's “National Science Challenges” | 2015 | Aotearoa/New Zealand | Māori communities | Qualitative exploratory study (Interviews) | To examine seemingly contradictory moves of the NSC failing to recognise Māori researchers yet noting interest in supporting knowledge derived from Māori experiences. | Māori advocacy in response leveraged its own rhetorical and institutional resources. |
| PsychiatrNews | Proposed health cuts in Reagan budget will harm American Indians, APA tells congress | 1983 | United States | American Indian communities | News article | To explain the implications of proposed budget cuts on Indigenous health. | President Reagan's Fiscal 1984 budget request will severely aggravate the already poor condition of health care provided to American Indians. |
| Ringer | For generations to come: exploring local fisheries access and community viability in the Kodiak Archipelago | 2016 | United States | Alaskan Native community | Dissertation | To understand what the loss of local fisheries participation and social capital means for the viability of Kodiak Archipelago fishing communities, people, economies, and cultural identities. | A paradigm shift is required to re-envision fisheries management that values and incorporates the social component into policy objectives and assessments without only prioritizing economic indicators. |
| Salmon | Aboriginal mothering, FASD prevention and the contestations of neoliberal citizenship | 2011 | Canada | Indigenous peoples in Canada | Essay | To examine the ways that neoliberal economic and political trajectories of Canadian state formation raise important questions about when, how and under what conditions colonial states support FASD prevention efforts among Indigenous peoples. | Linking FASD prevention to decolonisation efforts in the context of a neoliberal policy environment demands caution. While the neoliberalist impulse can assist communities in funding prevention campaigns, FASD is an expression of gendered and racialized conditions of disenfranchisement and abandonment. |
| Shorten | We need to turn the traditional notion of a fair go into a modern reality… The key will be to manage change with equity - 40 years of the Australian: Politics | 2004 | Australia | Aboriginal and Torres Strait Islander communities | News article | To argue for the need of a modern fair go that means not turning a blind eye. | The best decisions required for Australia's future will be based on the love of freedom, tolerance of difference, and an enduring sense of justice. |
| Soares et al. | Food Transition and Oral Health in Two Brazilian Indigenous Peoples: A Grounded Theory Model | 2019 | Brazil | Guarani and Kaingang communities | Qualitative exploratory study (Interviews) | To analyse the social representations of the Guarani and Kaingang peoples regarding the transformations in traditional food systems and effects on oral health. | The food transition experienced by the Guarani and Kaingang peoples was represented by the participants as a form of cultural discontinuity that affects aspects of the community identity and health. This phenomenon implies perceived health inequalities in oral status, high demand for treatment, and tension between Indigenous communities and biomedical healthcare systems. |
| Sobrado et al. | Küme mongen on the Coast: Contexts and Course Changes in Intercultural Health in the South of Chile | 2021 | Chile | Williche communities | Case Study | To analyse the relationship between intercultural health care and küme mongen (health, good health, or good life) in Williche territory. | The recovery of spaces for self-management in health care is part of that imagination, which to a certain extent materializes in the initiatives described and others like them. |
| Sotomayor & Barrero-Castillero | Globalization and vulnerable populations in times of pandemic: a Mayan perspective | 2020 | Mexico | Mayan Indigenous communities | Commentary | To explore the relationship between globalisation and COVID-19, from a Mayan perspective. | Providing protection, support, and equitable access to health care services for Indigenous communities in a culturally competent manner is imperative. |
| Stavig | Unwittingly agreed: Fujimori, neoliberal governmentality, and the inclusive exclusion of Indigenous women | 2021 | Peru | Peruvian Indigenous peoples | Essay | To investigate Fujimori’s neoliberal reformation of Peru and the overlap with the rise of global reproductive and sexual rights movement. | Through the National Program, the Peruvian state instrumentalized Indigenous women’s bodies to create a neoliberal citizenry. |
| Stephenson & Stephenson | The political ecology of cause and blame: environmental health Inequities in the context of colonialism, globalization, and climate change | 2016 | Canada | Haisla First Nation | Book chapter | To explore how similar tropes manifest themselves within environmental health. | Communities in vastly different geographic locations experience similar discursive environments where blame and responsibility for health problems, ranging from food insecurity and malnutrition to asthma and chronic respiratory illness, is attributed to local people’s behaviour and choices, while systemic factors are ignored. |
| Stienstra | Canadian Disability Policies in a World of Inequalities | 2018 | Canada | Indigenous peoples in Canada | Essay | To examine how settler-colonial relations have created embedded inequalities through neoliberalism which promotes self-investment and justifies cuts to social programs. | Material circumstances of women, men, girls, and boys with disabilities in Canada reflect underlying power relations based in disability, gender, and Indigenousness (among others); despite human rights protections, the competing ideas around disabilities reinforce neoliberal-ableism as well as a sacrificial citizenship by people with disabilities who see themselves as not productive |
| Susana Ramirez | Salud, globalización e interculturalidad: una mirada antropológica a la situación de los pueblos indígenas de Sudamérica | 2014 | Bolivia and Argentina | Mbyá-Guaraní, Chimanes, Moxeños, and Yuracarés Indigenous communities | Commentary | To reflect on the impact of globalisation and interculturalism on the living conditions of Indigenous peoples in South America. | The health of Indigenous people cannot be improved without environmental health. The institutions that “promote Indigenous health” are the same that support the destruction of their environment. The first action in promoting Indigenous health is not related to the provision of health care, but to the preservation of their land. |
| US Fed News | Health Costs of Globalization for Indigenous Peoples | 2007 | Bolivia | Amazonian Indigenous peoples | News article | To describe the link between maternal knowledge about plants and related health outcomes for Indigenous children. | Globalization threatens maternal plant knowledge which has poor health ramifications for children. |
| Warbrick et al. | The biopolitics of Māori biomass: towards a new epistemology for Māori health in Aotearoa/New Zealand | 2016 | Aotearoa/New Zealand | Māori | Qualitative exploratory study (Autoethnography) | To consider what impact biopolitics that creates a compliant self-governing weight-focused population has had on Māori health in Aotearoa/New Zealand. | We argue for a new epistemology that actively abandons the current biopolitics of ‘the health of Māori’s’ in favour of a new biopolitical future for Māori health, situated at the level of community. We see this example of an alternative discourse to neoliberal ideals also as a signpost for non-Māori health movements that are fed up with current health promotion and its undue focus on biomarkers such as weight. |
| Yashadhana et al. | Re-examining the gap: A critical realist analysis of eye health inequity among Aboriginal and Torres Strait Islander Australians | 2021 | Australia | Aboriginal and Torres Strait Islander communities | Qualitative exploratory study (Yarning) | To identify the factors that either constrain or enable accessibility of eye care for Aboriginal people living with diabetes in rural and remote communities in Australia, and to theorise mechanisms through which outcomes are generated, to propose solutions to address inequity. | Marginality is linked to structural factors that position Aboriginal culture as a barrier, and is reinforced through biomedical health systems, and the agents who operate in and influence them. To address eye health inequity, a shift in how Aboriginal cultural sovereignty is understood within health systems is needed, to position it as a strength that can facilitate eye care accessibility, and to support enhanced cultural responsivity among clinicians and service providers. |
| Young & Moses | Neoliberalism and Homelessness in the Western Canadian Arctic | 2013 | Canada | Inuvialuit, Gwich'in, and Métis communities | Mixed Methods (Community-based participatory research) | To explore the experiences of hard to house persons, and those working with them, regarding needs and gaps in services that may increase the likelihood of finding housing and improve levels of personal health, wellness, and security. | This research has shown that, at the local level, there is a great deal of community concern and energy being devoted to hard to house persons with mental illness and substance abuse problems, but these people have difficulty accessing services when needed. Front-line workers must be supported through increased funding and coordinated efforts. |
