# Supplementary material for: The impact of neoliberal generative mechanisms on Indigenous health: a critical realist scoping review
Source: Global Health. 2022 Jun 15;18:61. doi: 10.1186/s12992-022-00852-2 (PMC9199313; doi:10.1186/s12992-022-00852-2)
Supplement: Supplementary file 4 — Additional file 4. Generative mechanisms. [file 12992_2022_852_MOESM4_ESM.docx]

**GENERATIVE MECHANISMS**

| **Pathways** | **Generative Mechanisms** | **Core Principles of Neoliberalism** | **Health Condition** |
| --- | --- | --- | --- |
| Industrial exploitation of native natural resources had devastating effects leading to high levels of poisonous substances in the water and encouraged a fishing ban. This ban has had significant impact on the economic, social status and health of the Indigenous populations or communities utilizing the natural resource (Water) (Barges, 2008) | Exploitation and contamination of natural resources | Competitive and private markets | Poisoning |
| Industry is “changing our land and waters,” contamination of foods and land (Duran 2015) | Exploitation and contamination of natural resources | Competitive and private markets | General health |
| The construction of reservoirs and dams in the city of Misiones, Argentina introduced multiple diseases (Susana Ramirez, 2014) | Exploitation and contamination of natural resources | Competitive and private markets | General health |
| Mining is taking away habitats for traditional foods (caribou) (Duran, 2015) | Disruption of traditional foodways | Competitive and private markets | General health |
| Construction of transoceanic roads leads to irreversible damage to traditional lands (Susana Ramirez, 2014) | Exploitation and contamination of natural resources | Competitive and private markets | General health |
| Deterioration of the environment caused by pesticides, residuals from paper factories, contamination of water (Susana Ramirez, 2014) | Exploitation and contamination of natural resources | Competitive and private markets | General health |
| Loss of traditional foods and lands impacts mental wellbeing, a feeling termed “lonely for the ice” (Duran, 2015) | Interrupted relationship with traditional lands | Competitive and private markets | General health |
| The cultural meaning of land destruction impacts mental wellbeing and leads to increased use of alcohol, drugs and suicide rates (Susana Ramirez, 2014) | Interrupted relationship with traditional lands | Competitive and private markets | General health |
| Fishing bans led to disruption of ties with nature and classifying as asocial behaviour, which had an impact on the health of the Indigenous communities as well. (Barges, 2008) | Interrupted relationship with traditional lands | Competitive and private markets | Poisoning |
| The people were unable to cook and consume their traditional foods (fish) and sought to fatty, unhealthy "westernised" foods, leading to health problems (Barges, 2008) | Increased reliance on processed foods | Competitive and private markets | Poisoning |
| Neoliberal policies drafted the health reform policy in 1991, initiating privatization and promoting innovations; one of the innovations was not for profit organisations which were started to benefit the community but actually led to the inclusion of a business culture in healthcare system (Barnett & Bagshaw, 2020) | Healthcare systems and services do not reflect Indigenous cultures | Competitive and private markets | General health |
| Poor social conditions are reinforced by neoliberal policies which in turn affect the mortality and morbidity (Barnett & Bagshaw, 2020) | Increased social risk | Reduced public expenditure on infrastructure and social services | General health |
| Direct and indirect effects of the Global Financial (2008) crisis on the health services system; indirect effect was the social risk effect created by this crisis and the direct effect related to the reduction of health budgets leaving the public health services unprotected. (Barnett & Bagshaw, 2020) | Increased social risk | Reduced public expenditure on infrastructure and social services | General health |
| Government (neoliberalists and private organisations) claims of overinvestment in the health services but actually a decline was observed. This had an impact of the health (Barnett & Bagshaw, 2020) | Privatisation of healthcare | Competitive and private markets | General health |
| Combination of neoliberal government policies and racism which lead to inequitable health service provisions leading to disease favouring conditions (Briggs, 2002) | Inequitable provision of healthcare on the basis of race | Deregulation that facilitates economic activity and freedom of choice | Cholera |
| The manipulation of the cause and concept of this disease (unhygienic and unsanitary conditions); it was conveniently blamed on "Indigenous" lifestyle and conditions. This entire façade was conceptualised and executed by the government in an attempt to save face and maintain their global standing. (Briggs, 2002) | Blaming poor health outcomes on “Indigenous” lifestyles | Personal autonomy | Cholera |
| Neoliberal policies and decisions leading to increasing number of cuts in the Aboriginal Health organisations leading to limited research and implications on Aboriginal health (Browne& Stout, 2012) | Insufficient investment in Indigenous infrastructure | Reduced public expenditure on infrastructure and social services | General health |
| Western medicine and practices and disregarded the importance of Indigenous conceptions of Health. Indigenous indicators of health and wellbeing not recognised by western medicine (Brown et al 2012) | Failure to recognise and appreciate Indigenous knowledges and understandings of wellbeing | Personal autonomy | General health |
| Description of the intersection of components of the health care system and its connection with health of First Nations People (Brown et al 2012) | Failure to recognise and appreciate Indigenous knowledges and understandings of wellbeing | Personal autonomy | General health |
| The pathway describes the actual negative impact of neoliberal policies (privatisation) which is supposedly set up to benefit the vulnerable populations (Brown et al 2012) | Redistributive practices that harm Indigenous dignity and social wellbeing | Reduced public expenditure on infrastructure and social services | General health |
| An illustration from one of the Elders included in this study, which describes the connection of native languages and Health, in addition to the impact of neoliberalism which prevents them from using native languages. This imparts a sense of dispossession and effects the mental and spiritual health (Brown et al 2012) | Healthcare systems and services do not reflect Indigenous cultures | Competitive and private markets | General health |
| The connection of land to good health is described, along with the impact of western policies which are responsible for uprooting Indigenous peoples from their land. (Brown et al 2012) | Interrupted relationship with traditional lands | Competitive and private markets | General health |
| Cancer was attributed to the use of ch*e*micals and contamination to increase crop yields and increase market uptake, also genetically modified foods injected with hormones and antibiotics. (Dinorah Martinez et al. 2021) | Exploitation and contamination of natural resources | Competitive and private markets | General health |
| Urbanization, development, and lifestyle changes causes cancer. People favouring city jobs, leaving home. As life in city is expensive so women also work leaving kids unsupervised. Less stressful means of transport like buses as compared to walking to work which keeps you healthy. Both parents work so no time to cook and rely more on fast food and processed foods. More office jobs led to sedentary lifestyles. Urban developments like stoves, electric and water has decreased physical activity in the name of convenience. (Dinorah Martinez et al. 2021) | Forced acculturation and participation in the global economy | Competitive and private markets | Cancer |
| The use of bilingual interpreters/mediators for negotiations between Indigenous Peoples and the government can have severe consequences to the structure of Indigenous communities (Susana Ramirez, 2014). | Forced acculturation and participation in the global economy | Competitive and private markets | General health |
| Poor nutrition, rise of westernized diets and cultural changes or loss lead to unhealthy lifestyles attributing to rise of chronic diseases like cancer (Dinorah Martinez et al. 2021) | Increased reliance on processed foods | Competitive and private markets | Cancer |
| Globalisation has made calorie dense foods more available which leads to ill-health CVS disease (Dinorah Martinez et al. 2021) | Increased reliance on processed foods | Competitive and private markets | Nutrition |
| This illustration stresses on the relation between the market and being overweight. Due to increased availability and nutrition dense foods, it leads to weight issues in men and women. (Dinorah Martinez et al. 2021) | Increased reliance on processed foods | Competitive and private markets | Nutrition |
| Nutrition transitions as a result of industrialization, urbanization, economic development, and globalization (**Duran 2015)** | Increased reliance on processed foods | Competitive and private markets | General health |
| Neoliberal policies focus on individual responsibilities towards health and ignore the impact of the socio-economic and historic inequities. Non-compliance of the individual responsibility is tagged as shameful and blamed publicly for their failing health. (Durey et al. 2016) | Disregard for socioeconomic inequities and historic injustices experienced by Indigenous peoples | Personal autonomy | Oral health |
| Limited public dental services, long waiting lists, focus on treatment and not prevention. Private dental is out of reach (Durey et al. 2016) | Privatisation of healthcare | Competitive and private markets | Oral health |
| High cost of healthy diets and foods plus intense marketing of sugary products (Durey et al. 2016) | Increased reliance on processed foods | Competitive and private markets | Oral health |
| Neoliberal policies have advocated for socio-economic inequalities by introducing and reinforcing high living costs and poverty. These conditions in addition to food insecurities related to climate change are bound to introduce nutritional deficiencies, impacting the health (Ford, 2012) | Disregard for socioeconomic inequities and historic injustices experienced by Indigenous peoples | Personal autonomy | General health |
| Impact of globalisation in addition to past colonial trauma has reinforced the social and economic gaps (Ford, 2012) | Forced acculturation and participation in the global economy | Competitive and private markets | General health |
| A change from a traditional economy to a wage work economy has also contributed to the nutrition transition (Duran, 2015) | Forced acculturation and participation in the global economy | Competitive and private markets | General health |
| The upstream construction of the massive Lower Churchill hydroelectric development by NalCor, a provincial crown corporation presents a threat to water security with the additional part of water contamination due to industrial pollutants which manifests as health hazards to the local Indigenous communities (Hanrahan, 2017) | Exploitation and contamination of natural resources | Competitive and private markets | General health |
| The poor water conditions are due to poorly designed water plants, financial constraints etc. This gave rise to water insecurity. Neoliberal policies led to financial stresses which led to water insecurities and impacting health of Indigenous communities. (Hanrahan, 2017) | Exploitation and contamination of natural resources | Competitive and private markets | General health |
| The continuing impact of colonialism on Indigenous health by inducing water insecurities. The water insecurities are two-fold; 1. Lack of clean water - all water is contaminated by chemicals like mercury or micro-organisms like E.coli 2. by effecting the fishing produce (tourism burden) and mercury poisoning in fish - health hazards. (Hanrahan, 2017) | Exploitation and contamination of natural resources | Competitive and private markets | General health |
| Has referred to neoliberal policies as " social experimentation" which posed financial, social and health disparities amongst Indigenous populations of New Zealand (Hodgetts, 2004) | Increased social risk | Reduced public expenditure on infrastructure and social services | General health |
| Effect of extensive food sharing networks impact hunting and fishing practices of the local communities, which are directly or indirectly impacting the culture, nutrition, and health of the Indigenous communities. (Hovelsrud et al. 2011) | Disruption of traditional foodways | Competitive and private markets | General health |
| Climate change and increased tourism has impacted the purity of the natural resources which are extensively utilised by the local Indigenous communities. Lack of sanitation and increased pressure on the resources has an impact on health (more infections, impure water etc) (Hovelsrud et al. 2011) | Disruption of traditional foodways | Competitive and private markets | General health |
| Climate change is leading to increased thawing of ice and the level of pollutants in the water are increasing. Food is now contaminated and has disastrous impacts on traditional food and cultural aspects of the Indigenous populations, which highly rely on these natural resources. (Hovelsrud et al. 2011) | Disruption of traditional foodways | Competitive and private markets | General health |
| Neoliberal policies impact Indigenous health in two ways, firstly by medicalising consequences of the 'Indigenous disadvantage' and secondly, increased focus on the expression and presumed mediators of this disadvantage (violence, suicide, substance use, depression) (Hunter, 2020) | Disregard for socioeconomic inequities and historic injustices experienced by Indigenous peoples | Personal autonomy | Mental health |
| A description of the actual impact of the government policies which were intended to help Indigenous health (Jamieson, 2020) | Disregard for socioeconomic inequities and historic injustices experienced by Indigenous peoples | Personal autonomy | Oral health |
| Effect of Systemic racism introduced by neoliberal policies on Indigenous Health (Jamieson, 2020) | Inequitable provision of healthcare on the basis of race | Deregulation that facilitates economic activity and freedom of choice | Oral health |
| Impact on Indigenous oral health by neoliberal policies via increased wealth disparities (Jamieson, 2020) | Increased social risk | Reduced public expenditure on infrastructure and social services | Oral health |
| A detailed description of all the possible ways transnational corporations’ products and marketing impact the oral health and general health of Indigenous populations. (Jamieson, 2020) | Increased reliance on processed foods | Competitive and private markets | Oral health |
| Effect of Privatization of Health by incorporation of neoliberal policies on Indigenous Health (Jamieson, 2020) | Insufficient investment in Indigenous infrastructure | Reduced public expenditure on infrastructure and social services | Oral health |
| Increased dominance of transnational corporations without adequate regulation or oversight (Jamieson, 2021) | Increased reliance on processed foods | Competitive and private markets | Oral health |
| Privatisation of Healthcare (Jamieson, 2021) | Privatisation of healthcare | Competitive and private markets | Oral health |
| Domination of concepts of personal autonomy and responsibility (Jamieson, 2021) | Blaming poor health outcomes on “Indigenous” lifestyles | Personal autonomy | Oral health |
| The use of SRAs (Shared Responsibility Agreements) in place of health service provisions permits continuation of poor health (Lawrence & Gibson, 2007) | Inequitable provision of healthcare on the basis of race | Deregulation that facilitates economic activity and freedom of choice | General health |
| Assumption of rural Indigenous communities as 'failed' or 'ungovernable' spaces permits continuation of poor health (Lawrence & Gibson, 2007) | Blaming poor health outcomes on “Indigenous” lifestyles | Personal autonomy | General health |
| Pollution, globalisation of food system and forced uptake of dominant culture political system (neo)increases reliance on processed foods which increase occurrence of diet-related diseases (Liddell & Kingston, 2021) | Increased reliance on processed foods | Competitive and private markets | Reproductive health |
| Pollutants and toxins increased occurrence of fertility problems (Liddell & Kingston, 2021) | Exploitation and contamination of natural resources | Competitive and private markets | Reproductive health |
| Chronic health conditions in community because of pollution/toxins (Liddell & Kingston, 2021) | Exploitation and contamination of natural resources | Competitive and private markets | Reproductive health |
| Increased occurrence of cancer (Liddell & Kingston, 2021) | Exploitation and contamination of natural resources | Competitive and private markets | Reproductive health |
| Privatisation has influenced the articulation of new diets, leaving a strong mark on nutritional health (Lucas, 2017) | Increased reliance on processed foods | Competitive and private markets | Westernised diet |
| Disproportionate Covid-19 suffering due to limited access healthcare (Menton, 2021) | Inequitable provision of healthcare on the basis of race | Deregulation that facilitates economic activity and freedom of choice | Covid-19 |
| Mining of Indigenous territories considered an "essential service," development continued and miners brought Covid-19 into communities. (Menton, 2021) | Inequitable provision of healthcare on the basis of race | Deregulation that facilitates economic activity and freedom of choice | Covid-19 |
| Neoliberal reforms to mining law opened door to foreign investment which contaminates water, land, and increases violence (Moore & Moore, 2021) | Exploitation and contamination of natural resources | Competitive and private markets | General health |
| Under the guise of reconciliation, unsustainable neoliberal economics target Indigenous sources of food (Pictou, 2017) | Disruption of traditional foodways | Competitive and private markets | Indigenous food sovereignty |
| Limited funding to "close the gap" increasing poverty and hunger (Pictou, 2017) | Insufficient investment in Indigenous infrastructure | Reduced public expenditure on infrastructure and social services | Indigenous food sovereignty |
| Neoliberalism perpetuates patriarchal structures that limit Indigenous women's ability to protect their Lands and Waters (Pictou, 2017) | Perpetuation of colonial and patriarchal structures | Deregulation that facilitates economic activity and freedom of choice | Indigenous food sovereignty |
| Budget cuts leading to reduced health care available for Indigenous peoples (PsychiatrNews, 1983) | Inequitable provision of healthcare on the basis of race | Deregulation that facilitates economic activity and freedom of choice | General health |
| Privatisation of fishing means fishermen are considered self-employed and have no health care insurance (Ringer, 2016) | Increased social risk | Reduced public expenditure on infrastructure and social services | General health |
| Commodification of fisheries has decreased fishing opportunities, which has a negative impact on individual and community wellbeing (Ringer, 2016) | Interrupted relationship with traditional lands | Competitive and private markets | General health |
| Decreased youth involvement in fisheries because of privatisation has contributed to increased youth addiction/substance abuse (Ringer, 2016) | Interrupted relationship with traditional lands | Competitive and private markets | General health |
| Government spending focused on FASD prevention messaging rather than health service provision (Salmon, 2011) | Blaming poor health outcomes on “Indigenous” lifestyles | Personal autonomy | Foetal alcohol syndrome |
| Low life expectancy (Shorten, 2004) | Increased social risk | Reduced public expenditure on infrastructure and social services | General health |
| Participation in labour market requires consumption of industrialised foods (Soares, 2019) | Forced acculturation and participation in the global economy | Competitive and private markets | Oral health |
| Leasing of traditional lands decreases access to traditional foods (Soares, 2019) | Disruption of traditional foodways | Competitive and private markets | Oral health |
| Privatisation of health care prevented founding a health care centre until 2007 (Sobrado, 2021) | Insufficient investment in Indigenous infrastructure | Reduced public expenditure on infrastructure and social services | General health |
| Financial constraints force many Indigenous peoples to work in larger cities in the hotel industry, which increased exposure to Covid-19 (Sotomayor & Barrero-Castillero, 2020) | Forced acculturation and participation in the global economy | Competitive and private markets | Covid-19 |
| Limited access to healthcare heightened by Covid-19 (Sotomayor & Barrero-Castillero, 2020) | Privatisation of healthcare | Competitive and private markets | Covid-19 |
| Lack of pandemic response that reflects social, cultural, and historical context of pre-existing health disparities. (Sotomayor & Barrero-Castillero, 2020) | Failure to recognise and appreciate Indigenous knowledges and understandings of wellbeing | Personal autonomy | Covid-19 |
| Forced sterilization of Indigenous women to decrease poverty and increase economy (Stavig, 2021) | Perpetuation of colonial and patriarchal structures | Deregulation that facilitates economic activity and freedom of choice | Sterilisation |
| Industrial development of culturally inappropriate homes with inexpensive materials led to increased mold exposure (Stephenson & Stephenson, 2016) | Insufficient investment in Indigenous infrastructure | Reduced public expenditure on infrastructure and social services | Asthma |
| Public perception about disability and access to euthanasia increasing desire to die among those with a disability (Sienstra, 2018) | Association of worth or morality with health status | Personal autonomy | Disabilities |
| Neoliberal policies eroded mental health infrastructure and income assistance (Sienstra, 2018) | Increased social risk | Reduced public expenditure on infrastructure and social services | Disabilities |
| Commodification of care does not reflect Indigenous understandings of disability care (Sienstra, 2018) | Healthcare systems and services do not reflect Indigenous cultures | Competitive and private markets | Disabilities |
| Globalization threatens Indigenous knowledge related to poorer child health outcomes (US Fed News, 2007) | Failure to recognise and appreciate Indigenous knowledges and understandings of wellbeing | Personal autonomy | Child health |
| Prescription of weight loss, complicit with neoliberal biopolitics, disempowers individuals when unachievable (Warbrick, 2016) | Association of worth or morality with health status | Personal autonomy | Body weight |
| Neoliberalism sanitised the holistic view of Māori health (Warbrick, 2016) | Failure to recognise and appreciate Indigenous knowledges and understandings of wellbeing | Personal autonomy | Body weight |
| Biomedical neoliberal language leads to communication breakdowns and exclusion from eye health decision making (Yashadhana, 2021) | Healthcare systems and services do not reflect Indigenous cultures | Competitive and private markets | Eye health |
| ACCHS clinicians upholding neoliberal values that don't align with Aboriginal cultural systems (Yashadhana, 2021) | Blaming poor health outcomes on “Indigenous” lifestyles | Personal autonomy | Eye health |
| Lack of clinical Aboriginal leadership due to change in funding structures (Yashadhana, 2021) | Insufficient investment in Indigenous infrastructure | Reduced public expenditure on infrastructure and social services | Eye health |
| Neoliberal roll-back policies intensified housing shortage and obstructed development of addiction and mental health services (Young & Moses, 2013) | Insufficient investment in Indigenous infrastructure | Reduced public expenditure on infrastructure and social services | Mental health |

| **Resistance Pathway** | **Generative Mechanism of Resistance** | **Core principle of neoliberalism** |
| --- | --- | --- |
| Not a resistance pathway as pe Indigenous peoples directly, but the proposition of a resistance formed by the Nurses in Healthcare, which would help in offering better health care services for the Aboriginal populations (Brown et al 2012) | Ally advocacy for the protection of Indigenous rights | Deregulation that facilitates economic activity and freedom of choice |
| Environmental activists fighting to protect the health of tribal members (Liddell & Kingston, 2021) | Community resistance to industrial development | Competitive and private markets |
| Despite mandates to reduce support to activities that support Indigenous health during Covid, allies (such as Indigenist Missionary Council) continue to work to protect Indigenous rights (Menton, 2021) | Ally advocacy for the protection of Indigenous rights | Deregulation that facilitates economic activity and freedom of choice |
| Emergence of grassroots organisations and local resistance due to the inabilit of neoliberalism to provide answers (Menton, 2021) | Community resistance to industrial development | Competitive and private markets |
| Self-isolation and food sovereignty enabled communities to avoid Covid-19 exposure (Menton, 2021) | Utilisation of traditional practices to reduce reliance on neoliberal systems | Personal autonomy |
| Protests blocking access to mines demanding Covid-19 support from government (Menton, 2021) | Community resistance to industrial development | Competitive and private markets |
| Communities organising in peaceful resistance to protect land (Moore & Moore, 2021) | Community resistance to industrial development | Competitive and private markets |
| Profound resilience and teachings about alternative ways to live together with each other and with the very source of our survival: the natural ecosystems that sustain us (Indigenous women) (Pictou, 2017) | Utilisation of traditional practices to reduce reliance on neoliberal systems | Personal autonomy |
| Māori scholars advocated and overturned NSC decision to withdraw funding for Māori research centres (Prussing & Newbury, 2015) | Advocacy for increased investment in Indigenous health | Reduced public expenditure on infrastructure and social services |
| Subsistence farming to reduce reliance on industrialised products (Soares, 2019) | Utilisation of traditional practices to reduce reliance on neoliberal systems | Personal autonomy |
| Māori health movement in response to rise in neoliberalism (Warbrick, 2016) | Advocacy for increased investment in Indigenous health | Reduced public expenditure on infrastructure and social services |
| Aboriginal management of care strengthening Aboriginal health and limiting influence of neoliberal values on care (Yashadhana, 2021) | Utilisation of traditional practices to reduce reliance on neoliberal systems | Personal autonomy |

**RAW PATHWAYS DATA**

**Barges, 2008**

***Health Condition: Poisoning***

***Indigenous community: Ojibway, Anishinaabe, Cree (Grassy Narrows, White Dog, Mistassini/Noranda)***

| Pathway 1 Summary |
| --- |
| Industrial exploitation of native natural resources, had devastating effects leading to high levels of poisonous substances in the water and encoraged a fishing ban. This ban has had significant impact on the economic, social status and health of the Indigenous populations or communities utilizing the natural resource (Water) (Barges, 2008) |
| Pathway 1 Illustration |
| The pollution came from Chlorate-alkali factories that belonged to major industrial trusts producing pulp. As their land is accessible by roads, the Ojibway community has been exposed to non-native lifestyle for a much longer time than the Crees. The former experienced industrial exploitation of natural resources instead of tourism. In 1945 the natives ran the majority of commercial fishing companies, and the numerous fishing camps for tourists were their primary source of income. White Dog and Grassy Narrows communities had already suffered from two relocations that took place in 1956 and 1963 and brought about social and economic disruptions. Their lifestyle had been changing, but hunting and fishing remained important. In this province American Indian militancy emerged during these years. The territories of the Crees in Quebec, who mainly secured their livelihood by means of hunting and fishing, were not connected to roads for a very long time. Contact with the administration favouring settlement was only made in the 1970s, when projects of hydroelectric installations were carried out in the region. The dietary habits of those two communities are still very much based on fish. (Barges, 2008) |
| Pathway 2 Summary |
| Fishing bans led to disruption of ties with nature and classifying as asocial behaviour, which had an impact on the health of the Indigenous communities as well. (Barges, 2008) |
| Pathway 2 Illustration |
| Anthropologists have shown that the relationship with nature is vital. The human being is placed into animistic and holistic worlds, and intimate links exist with the entire ecosystem. The taxonomy of Algonquian distinguishes between forest animals, pillars of life and other animals. Hunting and fishing is a form of establishing mutual personal relationships between humans and animals. These animals contribute to the social tie, the stability of the group as well as to cultural stability. Disrespecting certain ties with nature is therefore considered as asocial behaviour, and everybody needs to take on responsibility for the balance of his/her Earth and his/her people. The territory is perceived as a calendar and coordinates these ties. The land of ancestors has also become a place of socio-economic relegation and has therefore reinforced the phenomenon of the inner circle Fish such as pike and walleye bream are some of the main ingredients in the traditional cuisine of these communities. Their style of cooking used to be rich in proteins, using only very little sugar or fat. An ideal meal (i.e., the most traditional and therefore the most valued meal) consists of game or fresh-water fish. Traditional meals favour commensality within the group. These meals are referred to as “genuine food”, which the community is confident in and which is described with native words. It is a pure form of food, with clear origins whose genesis can be traced back. European food means alteration or poisoning. The construction of the native body has a conflict-related relationship with western society, and in this context being disgusted means to resist. There is a political use of the body and food. However, inclusive food as an identity sign is also transforming. Flour and sugar have been appropriated for long. Even alcohol (Barges, 2008) |
| Pathway 3 Summary |
| The people were unable to cook and consume their traditional foods (fish) and sought to fatty, unhealthy "westernised" foods, leading to health problems (Barges, 2008) |
| Pathway 3 Illustration |
| The land of ancestors has also become a place of socio-economic relegation and has therefore reinforced the phenomenon of the inner circle Fish such as pike and walleye bream are some of the main ingredients in the traditional cuisine of these communities. Their style of cooking used to be rich in proteins, using only very little sugar or fat. An ideal meal (i.e., the most traditional and therefore the most valued meal) consists of game or fresh- water fish. Traditional meals favour commensality within the group. These meals are referred to as ‘‘genuine food’’, which the community is confident in and which is described with native words. It is a pure form of food, with clear origins whose genesis can be traced back. European food means alteration or poisoning. |

**Barnett & Bagshaw, 2020**

***Health Condition: General health***

***Indigenous community: Maori***

| Pathway 1 Summary |
| --- |
| Neoliberal policies drafted the Health reform policy in 1991, initiating privatization and promoting innovations; one of the innovation was Not for profit organisations which were started to benefit the community but actually led to the inclusion of a business culture in healthcare system (Barnett & Bagshaw, 2020) |
| Pathway 1 Illustration |
| The new National government (1990–96) pressed on with neoliberal policies. Severe cuts to welfare occurred in 1991 along with legislation to deregulate and create a more ‘efficient’ labour market. The aim of health reform policy (1991) was to increase effi ciency through privatisation and a competitive market, and opportunities for innovation. The development of not-for-profit (NFP) organisations providing health services to Māori, Pasifika, youth and mentally ill people were important innovations to benefi t the community, but otherwise there was a retreat from the excesses of the 1991 policy, first under a coalition government (1997–99) and a Labour government 2000–2009. Nevertheless, as the theory and practice of the New Public Management became consolidated in the 1990s, its business culture became embedded in the health system where it still pervades language and the planning and delivery of services |
| Pathway 2 Summary |
| Poor social conditions are reinforced by neoliberal policies which in turn affect the mortality and morbidity (Barnett & Bagshaw, 2020) |
| Pathway 2 Illustration |
| Research worldwide has shown how poor health outcomes are related to deterioration in the social and economic determinants of health, such as income, housing, food security, employment, stress and educational opportunities. Poor social conditions are not accidental, but result from neoliberal policies that affect not only mortality but also morbidities such as obesity, mental health and health risk behaviours. In New Zealand these relationships have been demonstrated over decades, with health risks from neoliberalism borne disproportionately by Māori and Pacifi c people19 and exacerbated by the experience of cultural loss, colonisation and racism |
| Pathway 3 Summary |
| Direct and indirect effects of the Global Financial (2008) crisis on the health services system; indirect effect was the social risk effect created by this crisis and the direct effect related to thereduction of health budgets leaving the public health services unprotected. (Barnett & Bagshaw, 2020) |
| Pathway 3 Illustration |
| Besides the poor economic performance associated with austerity, there is also a negative relationship between austerity and health. First, as already discussed, is the ‘social risk effect’, or ‘risk-shifting’ where those already disadvantaged bear the consequences of deterioration in the determinants of health. The second is through the direct impact on health services. For example, after the Global Financial Crisis of 2008, health outcomes for countries where health budgets were reduced compared unfavourably with countries that protected spending on public services.23 In New Zealand, health service impact is caused by both persistent underinvestment in services, competitive approaches and the marginalisation of health professionals in decision-making. |
| Pathway 4 Summary – may not be a pathway |
| Government (noeliberalists and private organisations) claims of overinvestment in the health services but actually a decline was observed. This had an impact of the health (Barnett & Bagshaw, 2020) |
| Pathway 4 Illustration |
| Government claims of overfunding in health services in the late 1980s were rejected by professional economists, but the narrative of ‘overfunding’ was reinforced by self-interested private organisations and neoliberal governments. In fact, there is no indication of unsustainable funding between 2000–2015. Measures of health expenditure for 2009–2018, adjusted for infl ation and population change, based on Treasury models, indicated a cumulative decline. The result was an effective reduction in funding for health services. |

**Briggs, 2002 – S noted to read this paper**

***Health Condition: Cholera - disease***

***Indigenous community: Warao***

| Pathway 1 Summary |
| --- |
| combination of noeliberal governmaent policies and racism which lead to inequitable health service provisions leading to disease favoring conditions (Briggs, 2002) |
| Pathway 1 Illustration |
| a |
| Pathway 2 Summary |
| The manipulation of the cause and concept of this disease (unhygeinic and unsanitary conditions); it was conveniently blamed on "Indigenous" lifestyle and conditions. This entire façade was conceptualised and executed by the governemnt in an attemptto save face and maintain their global standing. (Briggs, 2002) |
| Pathway 2 Illustration |
| The devastating effects of the epidemic continued to be felt long after it was officially declared over in the middle of 1993. Faith in vernacular healing was undermined, and institutional physicians and their medicines, particularly antibiotics, came to be seen by many delta residents as possessing magical powers. After the cholera scandal had passed, the impressive emergency infusion of physicians, medicines, boats and gasoline disappeared. The cholera epidemic and the subsequent exodus of Mariusans and their neighbors to major cities had discomfited the state government, threatened its legitimacy, and further stigmatized Delta Amacuro as being a bastion of backwardness and ignorance, a premodern cancer on a modernizing country. "The Warao" were seen not simply as an embarrassment and an obstacle to exploitation of the delta's resources, but as a political liability. Therefore, the few clinics established in this vast area were often without even aspirin on their shelves. When patients were turned away by disillusioned physicians and nurses, institutional medicine was also delegitimated. "When they wanted to save our lives, they did," noted one delta resident. "Now they want us to die."  Race and class clearly lie at the core of these fears. The story of the cholera epidemic in Delta Amacuro is not a simple tale of Machiavellian conspiracies or evil powermongers who gleefully marked others for death. It is, rather, a story of well-trained professionals who, in general, took their obligation to protect the health of the public quite seriously. It is not a tale of a backward, Third World country in which callous officials were ignorant of or unconcerned with modernizing health care. The citizens of oil-rich Venezuela have long prided themselves on being a shining example of democracy and modernity in Latin America. Moreover, the denigrating images and timeworn stereotypes attached to the epidemic were not invented in Venezuela alone. Medicines, techniques of diagnosis and treatment, technologies, manuals, statistics, reports, and interpretations are transnational, moving rapidly among public health institutions around the world. |

**Browne & Stout, 2012**

***Health Condition: General health***

***Indigenous community: First Nations, Inuit, and Métis***

| Pathway 1 Summary |
| --- |
| Neoliberal policies and decisions leading to increasing number of cuts in the Aboriginal Health organisations leading to limited research and implications on Aboriginal health (Browne& Stout, 2012) |
| Pathway 1 Illustration |
| In their Discourse contribution, David Gregory and Jean Harrowingdiscuss the sweeping cuts to Aboriginal health organizations now occur-ring in Canada that will have serious implications for communities andthe kinds of sustained research partnerships that are necessary to addresshealth priorities at the local and population levels. These cuts will leavelarge holes to fill, and are part and parcel of the neoliberal political ide-ologies that are accelerating health and social inequities in Canada andglobally. |

**Brown et al. 2012**

***Health Condition: General health***

***Indigenous community: Namg First Nation***

| Pathway 1 Summary |
| --- |
| Western medicine and practices and disregarded the importance of Indigenous conceptions of Health. Indigenous indicators of health and wellbeing not recognised by western medicine (Brown et al 2012) |
| Pathway 1 Illustration |
| Some authors use the term “medical colonialism” to describe the tendency of Western medical practices and conceptions of health to undermine the integrity of Indigenous communities, and, in Kelm’s (2004) words, “to disrupt Indigenous social structures as a way of enforcing acculturation”. In the current health-care context, neoliberal ideologies discourage us from addressing threats to cultural integrity and the complexities of historical trauma, and tempt us to interpret health challenges in Aboriginal communities as decontextualized individual problems. As a result, the solid evidence on the social determinants of health has minimal impact on policies and practices, and the place of recovering culture and identity as the “social capital” crucial to improving the health of Indigenous people tends to be absent from health policy and planning priorities. |
| Pathway 2 Summary |
| Description of the intersection of components of the health care system and its connection with health of First Nations People (Brown et al 2012) |
| Pathway 2 Illustration |
| The ability to see the ways in which multiple and intersecting forms of dispossession accumulate and lead into spaces of deprivation and exclusion may help nurses to comprehend how historical colonial relations take neocolonial forms and are manifested in the health and well-being of Indigenous people today. This perspective brings to light the fact that the people, structures, practices, and policies within health care actually shape and create the spaces where First Nations experience health care. Sparke (2007) claims that any repossession of space for a better world, such as one where health equity can be realized, calls for an examination of “space-making processes and space-framing assumptions” |
| Pathway 3 Summary |
| The pathway describes the actual negative impact ofneoliberal policies (privitization) which is supposedly set up to benefit the vulnerable populations (Brown et al 2012) |
| Pathway 3 Illustration |
| Harvey (2005) is also unequivocal about the harms caused by the economic sequelae of neoliberal policies: “the redistributive tactics of neoliberalism are wide ranging, sophisticated, frequently masked by ideological gambits but devastating for the dignity and social well-being of vulnerable populations and territories”. MacDonald (2009) argues that while achieving Indigenous self-governance might appear to benefit from neoliberalism’s politics of privatization through rejection of state interventions known to undermine Aboriginal autonomy and self- determination  in fact vulnerable populations face |
| Pathway 4 Summary |
| An illustration from one of the Elders included in this study, which describes the connection of native languages and Health, in adddition to the impact of Neolib/westernisation which prevents them from using native launguages. This imparts a sense of dispossesion and effects the mental and spiritual health (Brown et al 2012) |
| Pathway 4 Illustration |
| "Our language is our culture; it is the medium, or the form, or the process, that allows us to give full expression to who we are, mentally, physically, spiritually, collectively, as friends and family, individually, historically [and] looking forward. It’s the only medium we have that can do that. As long as we have our mind-set we’re not going to be struggling with Western concepts [like] what’s right or wrong. The creator never intended that to be the way it is. We’re Kwakwaka’wakw and he gave us laws that are spiritual, that will sustain us through time. We will be the healthiest when we can give expression to that. |
| Pathway 5 Summary |
| The connection of land to good health is described, along with the impact of wetsern policies which are responsible for uprroting Indigenous peoples from their land. (Brown et al 2012) |
| Pathway 5 Illustration |
| Several Elders associate displacement and the reserve system as severing spiritual connections that affect the procurement of traditional foods and the diet that today is associated with poor health: I think back to when we lived in Karleqwes. You know, we lived off the land . . . we had all the fresh salmon, clams, mussels, crabs, fresh deer when the season would come around. Now I found out that when we moved out of Karleqwes it’s the first time that I really got sick, and, you know, could never understand why I was going through all that as a kid. The doctors couldn’t do anything. Mom did everything to get me better, and the funny thing too is that when I first came out here I couldn’t eat hamburger or beef and I couldn’t eat Kraft dinner [laughing], but I found that if we could make use of our old village and just go back there and do our harvesting and stuff like that . . . |
| Resistance Pathway 1 Summary |
| Not a resistance pathway as pe Indigenous peoples directly, but the proposition of a resistance formed by the Nurses in Healthcare, which would help in offering better health care services for the Aboriginal populations (Brown et al 2012) |
| Resistance Pathway 1 Illustration |
| By resisting the “naturalization” of language decline and disconnection from traditional territories, nurses can orient their actions towards creating opportunities for repossession as a fundamental dimension of health-promoting care for Indigenous people. Nurses can denaturalize “losses” through colonization and can engage with Indigenous people, knowing how dispossession causes disconnection and oppression and contributes to health and health-care inequities. Curiosity about the connections between identity, land, language, and health could be understood as a useful component of health assessment. |

**Dinorah Martinez et al. 2021**

***Health Condition: Cancer***

***Indigenous community: Kichwa***

| Pathway 1 Summary |
| --- |
| Cancer was attributed to the use of ch*e*micals and contamination to increase crop yields and increase market uptake, also genetically modified foods injected with hormones and antibiotics. (Dinorah Martinez et al. 2021) |
| Pathway 1 Illustration |
| P6: The chemicals they put in the food/what we eat and (got cut off) P6: On the vegetables and fruits P1: Like I was saying, they put them so they grow faster and now they fumigate and add more, and then everything grows really quickly and that is also causing a lot of harm to people I think. P4: In general, to all of us. P8:They give [you] diseases like cancer P2: I think it is the chemicals, because you know, my grandmother says this ‘Now we buy potatoes, we buy that and then we get cancer and before it was not like this.’ Then, realistically, I think it is from that because before we ate much mor organically and now they put that on all the vegetables, legumes and all the food that we consume, everything is full of chemicals. |
| Pathway 2 Summary |
| Urbanization, development and lifestyle changes causes cancer. People favoring city jobs, leaving home. As life in city is expensive so women also work leaving kids unsupervised. Less stressful means of transport like buses as compared to walking to work which keeps you healthy. Both parents work so no time to cook and rely moreon fast food and processed foods. More office jobs led to sedentary lifestyles. Urban developments like stoves, electric and water has decreased physical activity in the name of convenience. (Dinorah Martinez et al. 2021) |
| Pathway 2 Illustration |
| Participants also attributed the rise in cancer to development/urbanization and lifestyle change that came along with it. They shared how many people in their community were leaving agricultural work in favor of city jobs. Men, for example, are finding jobs in construction. Where traditionally the women stayed home to take care of the children, now more women are working outside the home – finding work in the nearby flower houses/nurseries or as domestics/ housekeepers/caretakers in the city centers. Thus, dual income households can now afford to purchase more commodities and household goods. Instead of walking to work many now take cars/buses since they have a longer commute. With both parents working, families now rely more on processed and fast foods that are easy/quick to prepare. Participants also stated that families ate less home cooked meals in part because women are now working outside the home. In addition, the shift in work from more agricultural areas to urban centers has created more sedentary lifestyles with people walking less. Also, some of the technological advancements that come with development such as electricity, water in the house, and gas stoves have decreased physical activity and also brought perceived contaminants into the home. Participants also discussed changes in how land and personal/family plots are now being used to grow food to sell instead of for the family's own subsistence. |
| Pathway 3 Summary |
| Poor nutrition, rise of westernized diets and cultural changes or loss lead to unhealthy lifestyles attributing to rise of chronic diseases like cancer (Dinorah Martinez et al. 2021) |
| Pathway 3 Illustration |
| The majority of participants consistently made a connection between cancer and alimentación (nutrition) which is closely interrelated with the aforementioned themes. Alimentación was defined by the community as the food that one consumes. The rise in cancer was also attributed to mala alimentacion/desnutricion (poor/bad nutrition) and increased preference for western foods such as noodles, bologna, processed foods and fried food (e.g. fries) over traditional foods such as corn tostada, fava beans, hominy and meat. Also discussed were children's preferences for western foods and sweets over more traditional home cooked meals especially in the school setting |

**Donders & Barriocanal, 2020**

***Health Condition: Nutrition and chronic disease***

***Indigenous community: Tsimane,’ Huaorani***

| Pathway 1 Summary |
| --- |
| Globalisation has made calorie dense foods more available which leads to ill-health CVS disease (Dinorah Martinez et al. 2021) |
| Pathway 1 Illustration |
| 1. An increased intake in calorically dense foods (e.g., refined sugars, saturated fats) is typical for the nutritional transition and leads to obesity and nutrition related noncommunicable diseases (NR-NCDs), such as cardiovascular disease. 2. Increased accessibility to a physical marketplace is an important change in the food environment of hunter-gatherers. It impacts dietary choice by improving access to purchased foods including fats and sweets, while reducing access to nutritionally important foods from traditional sources |
| Pathway 2 Summary |
| This illustration stresses on the relation between the market and being overweight. Due to increased availibility and nutrition dense foods, it leads to weight issues in men and women. (Dinorah Martinez et al. 2021) |
| Pathway 2 Illustration |
| These findings are valid on a macro-level, but do not seem to consider the importance of local dynamics such as rapid economic changes. In addition to the findings of Wells et al., Rosigner et al. state that in such situations, changes in body composition, such as the likelihood of being overweight and an increase in BMI, are related to market expenditures. They show stronger results for men, due to the fact that mostly men are participating in local markets, which increases the access to market foods. Women’s activity levels related to food production are therefore not reduced in the same way as men’s when the levels of market involvement increase. |

**Duran, 2015**

***Health Condition: General health***

***Indigenous community: Dene First Nation***

| Pathway 1 Summary |
| --- |
| Nutrition transitions as a result of industrialization, urbanization, economic development, and globalization (**Duran 2015)** |
| Pathway 1 Illustration |
| Indigenous peoples of the Arctic are experiencing nutrition transitions as documented by the decreasing percentage of Indigenous peoples consuming Traditional diets (Kuhnlein, Receveur, Soueida, & Egeland, 2004). Changes in the accessibility and distribution of wildlife species as a result of climate change, along with social, cultural, economic, and political changes in the Arctic, have contributed to these nutrition transitions (Anisimov et al., 2007). When Arctic Indigenous peoples lose access to traditional foods, their nutrient intakes and subsequent health outcomes experience a variety of impacts. Traditional diets are low in fat, high in protein, and high in nutrients. Additionally, harvesting traditional foods contributes to physical fitness (Damman et al., 2008). The reduced intake of traditional foods among Indigenous peoples may result in reduced dietary intake of protein, iron, zinc, vitamin C, and fiber (Guyot et al., 2006). For example, the reduced intake of berries may result in reduced Vitamin C intake. But the greater concern with the reduced intake of traditional food is the transition to a Westernized diet, which is more likely to result in an increase in obesity (Damman et al., 2008). Traditional foods may be replaced with high-fat, high-calorie, and nutrient-empty market food (Kuhnlein et al., 2004). A reliance on market food may be a coping strategy in response to the changes associated with climate change, but it has serious consequences for Indigenous peoples. |
| Pathway 2 Summary |
| Industry is “changing our land and waters,” contamination of foods and land (Duran 2015) |
| Pathway 2 Illustration |
| Participants are aware that climate change only partially explains the changes to their environment. Contamination and changes in the migration patterns of caribou are also attributed to economic development projects in the region. Participants were concerned about the environmental impacts of nearby mining efforts for gold and diamonds, as well as the effect the tourism and outfitters industries had on the environment. Additionally, the contamination of the land and water, including snow, concerned participants: “I find that even the snow too (has changed), before it used to be three or four feet of snow but every time we set up a tent out on the land you only see maybe 12 to 18 inches of snow. We still melt the snow that is still out there. We are lucky to have that. It’s all polluted through [by] the mine or through all this mining, oil and different companies coming in and cutting timbers and all that.” “People are cautious now. About the food intake and where they should get it. At one time we would get all of our traditional food from Yellowknife Bay and from around our communities. We used to put a net out in front of the communities, we used to get the birds and the fish. But now we can’t do that no more. We really believe that our food is contaminated because of the city growth and mining growth, tailing ponds, sediments and so on.” |
| Pathway 3 Summary |
| A change from a traditional economy to a wage work economy has also contributed to the nutrition transition (Duran, 2015) |
| Pathway 3 Illustration |
| The majority of wage work requires employees to work Monday through Friday, and limits the time that Indigenous peoples have to go on the land and harvest. Social changes, such as Indian Residential Schools, have also contributed to the loss of traditional skills and knowledge necessary to harvest traditional foods (Quinn, 2007). |
| Pathway 4 Summary |
| Mining is taking away habitats for traditional foods (caribou) (Duran, 2015) |
| Pathway 4 Illustration |
| “It’s not like before. Remember the first time I went there [Barren Land]? Before the mines came there were lots of caribou tracks, now you don’t even see any. Mines are right on the migration paths, stops the caribou from coming down.”  “So the animals, it’s they the animals for as long as they live they go to the same route, same places, back and forth, go north have their young, come back [to North] in November December, come back in March so people could use it, they come back, but in their way are developers. Developers are in their way, so they [animals] go and change routes.”  “The way you look at it now, all the mines started it. All the animals we traditionally hunted are all gone now. Just all mines now. All the animals are making different routes now. Before I was used to having lots of caribou before all that mine stuff. Caribou not as much now... There’s another mine they want to open and in our hunting area. The moose are there. When that mine starts there’s going to be nothing because all the animals will move, will migrate again with all the blasting.”  The growth of the mining industry and the need for labor comes with an expansion of the city, both in terms of its infrastructure and its population growth. Northern residents also had access to hunting tags. Participants recognize that these factors combined are impacting the health and size of the caribou herd:  “One year there was so much caribou, people were just shooting caribou all around here. Since then caribou numbers come down. It’s hard to control people. I see lots when I was out on the land trapping. It’s not all us. It’s some people [non-Aboriginal] in town eat wild meat too, they shoot caribou.” |
| Pathway 5 Summary |
| Loss of traditional foods and lands impacts mental wellbeing, a feeling termed “lonely for the ice” (Duran, 2015) |
| Pathway 5 Illustration |
| An Elder succinctly described the sentiment, shared among participants, of longing for a simpler time on their land. This nostalgia was paired with a keen awareness that the remediation of the land and waters takes time: “I’d rather, I’d rather um you know I wish I could turn the clock back. I wish. What I see today I wish I could turn the clock back of [on] all the mining that you know that oil spill, the oil, and all over our country you know. I don’t know if things are ever going to change back. It’ll take a long time.” |

**Durey et al. 2016**

***Health Condition: Oral health***

***Indigenous community: Aboriginal***

| Pathway 1 Summary |
| --- |
| Neolibral policies focus on individual responsibilities towards health and ignore the impact of the socio-economic and historic inequities. Non-compliance of the individual responsibility is tagged as shameful and blamed publically for their failing health. (Durey et al. 2016) |
| Pathway 1 Illustration |
| The current neoliberal political and economic climate has increasingly focused on individual responsibility to make optimum health and lifestyle choices. This would include individuals making optimum oral health choices by regular attendance at the dentist for check-ups, eating healthy, sugar free food and brushing and fl ossing regularly. Penalties for ‘non-compliance’ with such public health messages to stay healthy are ‘blame’ and ‘personal failure’ . This neoliberal focus on individual responsibility for health reproduces the discourse that Aboriginal people are seen as the problem for not making good oral health choices or, if they see a dentist, not complying with expert advice to improve their oral health. What is avoided in this discourse are the socioeconomic and historic inequities impacting on Aboriginal people’s lives and collective experiences of discrimination including in healthcare. |
| Pathway 2 Summary |
| Limited public dental services, long wating lists, focus on treatment and not prevention. Private dental is out of reach (Durey et al. 2016) |
| Pathway 2 Illustration |
| Findings indicated opportunities for accessing care were constrained by limited availability of public dental services for Aboriginal adults, often with long waiting lists or waiting times where the focus of care was on treating rather than preventing disease. Use of services was constrained by cost with private services generally out of reach fi nancially and public services often incurring a co-payment. Aboriginal participants’ perceptions that they were discriminated against by health providers also reduced access. Findings also indicated that despite evidence of poor oral health for Aboriginal Australians, demand for services was not met by supply; the system of oral health care in Western Australia is mainly a private model of treatment with limited public oral health services including those related to education on prevention |
| Pathway 3 Summary |
| High cost of healthy diest and foods plus intense marketting of sugary products (Durey et al. 2016) |
| Pathway 3 Illustration |
| Despite the importance of oral health, preventing oral disease was also constrained by high cost of healthy diets on limited budgets and intense marketing of sugary products. This often led to participants accessing dental services for treatment not prevention, oral health being compromised by little or no education on preventing disease and promoting health. |

**Ford, 2012**

***Health Condition: General Health***

***Indigenous community: Indigenous peoples globally, with a focus on Inuit, Amazonians, Alaskan Natives***

| Pathway 1 Summary |
| --- |
| Neolibreral policies have advocated for socio-economic inequalities by introdicuing and reinforcing high living costs and poverty. These conditions in addition to food insecurities related to climtae change are bound to introduce nutritional deficiencies, impacting the health (Ford, 2012) |
| Pathway 1 Illustration |
| Among Inuit populations, a high prevalence of food insecurity associated with poverty and high cost of living is expected to increase sensitivity to nutritional deficiency caused by climate change---related stresses on traditional food systems, with women and children identified to be at high risk; similar challenges have been identified for Alaska Native villages, and among Amazonian communities. |
| Pathway 2 Summary |
| Impact of globalisation in addition to past colonial trauma has reinforced the social and economic gaps (Ford, 2012) |
| Pathway 2 Illustration |
| There is concern expressed in the literature that social, economic, and political transformations—rooted in colonial history, assimilationist policies, and accelerated by globalization—are having implications, many negative, for indigenous populations and their adaptive capacity. Poverty, marginalization, and lack of legal land title are all important contextual factors that affect adaptation and will be key challenges in the future. In Alaska and northern Canada, transformations of identity and community have resulted in loss of control, cultural continuity, and a weakening of traditional knowledge systems and social capital. Notwithstanding these historical conditions, significant progress has been made in developed nations in settling land claims and recognizing indigenous title, national governments have apologized for the colonial practices of history (e.g., Canada, Australia), and devolution of power to indigenous institutions has occurred in some jurisdictions. These developments hold promise for addressing some of the broader determinants of socioeconomic and health inequality that lie at the heart of indigenous vulnerability to climate change. Poorer nations face more substantial challenges. Particularly in remote regions experiencing significant resource development pressure, indigenous integration into the global economy is occurring often with limited respect for local autonomy, and accompanied by social and political violence. Associated acculturation challenges have been linked to problems of addiction, suicide, and a weakening of social networks and traditional knowledge systems that play an important role in indigenous health systems and contribute to significant adaptive capacity. |

**Hanrahan, 2017**

***Health Condition: General health***

***Indigenous community: Nunatsiavut Inuit***

| Pathway 1 Summary |
| --- |
| The upstream construction of the massive Lower Churchill hydroelectric development by NalCor, a provincial crown corporation presents a threat to water security with the additional part of water contamination due to industrial pollutants which manifests as health hazards to the local Indigenous communities (Hanrahan, 2017) |
| Pathway 1 Illustration |
| The Nunatsiavut Inuit community of Rigolet, Labrador is one of many Circumpolar Indigenous communities grappling with climate change and decreasing water levels. Rigolet’s drinking water system consists of municipal tap water, bottled water that is purchased locally and water collected from ‘running brooks’. This last source is consumed when Inuit engage in land-based activities, such as hunting, but it is also brought back to people’s homes for consumption. Decreasing water levels have myriad environmental, health and financial implications, such as increased financial barriers and decreased access to preferred water sources. In the case of Rigolet and other Indigenous communities in the area, the upstream construction of the massive Lower Churchill hydroelectric development by NalCor, a provincial crown corporation, may present another threat to water security. |
| Pathway 2 Summary |
| an example of the poor water conditions affecting another Indigenous community. The poor water conditions are due to to poorly designed water plants, financila constraints etc. This gave rise to wtaer insecurity. So we can say neoliberal policies led to financial stresses which led to water insecurities and impacting health of Indigenous communities. (Hanrahan, 2017) |
| Pathway 2 Illustration |
| In 2005 Kashechewan was temporarily evacuated after E. coli bacteria was found in its water supply, supported by a treatment site run by inadequately trained operators. But ‘over a year later, Kashechewan … continue[d] to struggle with poorly designed water plants or overly modern [meaning complicated] systems considered too costly to staff or maintain’; water crises continued. In January 2007, 21 people, including a nine-year-old, attempted suicide. While it would be disrespectful to pry into the suffering of others, it has to be noted that the people of Kashechewan, Ontario have multiple multi-generational losses and squalid living conditions bearing down on them, as do the people of Black Tickle, Labrador, and many other Indigenous communities. Water insecurity runs through the complex grid that makes up contemporary Canadian colonialism. |
| Pathway 3 Summary |
| This pathway directly quotes the continuing impact of colonialism on Indigenous health by inducing water insecurities. The water insecurities are two-fold; 1. Lack of clean water - all water is contaminated by chemicals like mercury or micro-organisms like E.coli 2. by effecting the fishing produce (tourism burden) and mercury poisioning in fish - health hazards. (Hanrahan, 2017) |
| Pathway 3 Illustration |
| First Nations reserves in or near urban areas are not immune to serious water insecurity; thus remoteness is not a factor in at least some of the water disparities in Canada. Bkejwanong (Walpole Island) First Nation, home to Anishnaabe, Potawatomi and Ottawa peoples, is located downstream from ‘Chemical Valley’, an area of Ontario with a high concentration of petrochemical industry. A 2010 study found that residents identified water pollution as a chief health concern due to the community’s proximity to Chemical Valley. Not surprisingly, First Nations citizens worried about the health status of children and elders and their stress was manifested in high cortisol levels in hair samples. Eighteen per cent of local fish sampled contained mercury at rates that exceeded the Ontario guideline limit. These case studies demonstrate that programmes such as the First Nations Water Management Strategy, which saw an investment of $600 million dollars on-reserve, obviously failed to meet one of its three objectives: ‘Water standards will be met by all facilities’ (Indigenous and Northern Affairs Canada 2010). It is important to note that the Strategy included reserves only; non-status First Nations, Inuit, and Métis communities were excluded. The two Inuit case studies here point to significant water security needs in Inuit communities. In addition, the three reserve case studies identify problems as serious as the presence of E. coli and mercury in water supplies. At Bkejwanong (Walpole Island) First Nation, colonialism through water insecurity continues to impact directly upon Indigenous bodies, as residential schools and other policies and programs have done in the past |

**Hodgetts, 2004**

***Health Condition: General health***

***Indigenous community: Maori***

| Pathway 1 Summary |
| --- |
| Has referred to neolibreal policies as " social experimentation" whichs posed financial, social and health disparities amongst Indigenous populations of New Zealand (Hodgetts, 2004) |
| Pathway 1 Illustration |
| As a result of this social experimentation there is now wider acceptance that factors such as reduced income, increased housing costs and social fragmentation undermine social capital and impact negatively on health. For instance, the disparities report provides compelling evidence for growing gaps between Maori and non-Maori health being associated with growing gaps between ‘the haves’ and ‘the have nots’. As the report shows, Maori life expectancy increased steadily through the 1950s to 1970s. However, in the early 1980s, when social reforms were introduced, it plateaued while Pakeha life expectancy continued to increase. As a result, the gap between Maori and non-Maori life expectancy increased from 7 to 10 years over the 1980s and 1990s. Poverty is associated with health. And more than this, whatever our socioeconomic position, we are likely to be experiencing worse health than the group who is a little better off than we are—in terms of education, occupation, income or deprivation. Action to reduce inequalities in health, therefore, has the potential to improve the health of all New Zealanders. In New Zealand, ethnic identity is an important dimension of health inequalities. Maori health status is demonstrably poorer than other New Zealanders; actions to improve Maori health also recognise Treaty of Waitangi obligations of the Crown. |

**Hovelsrud et al. 2011**

***Health Condition: General health***

***Indigenous community: Not specific, but mention Inuit and Sami***

| Pathway 1 Summary |
| --- |
| Effect of extensive food sharing networks impact hunting and fishing practices of the local communities, which are directly or indirectly impacting the culture, nutrition and health of the Indigenus communties. (Hovelsrud et al. 2011) |
| Pathway 1 Illustration |
| Hunting and fishing activities are important for nutrition, health, and household and community economies, and also have great cultural value in many indigenous and local communities. Because of extensive food-sharing networks, changing sea-ice conditions that have an impact on hunting will also affect those not directly involved in hunting |
| Pathway 2 Summary |
| climate change and increased tourism has impacted the purity of the natural resources which are extensively utilised by the local Indigenous communties. Lack of sanitation and increased pressure on the resources has an impact on health (more infections, impure water etc) (Hovelsrud et al. 2011) |
| Pathway 2 Illustration |
| Concern is also raised regarding the possibility of increased rates of foodand water-borne diseases and respiratory infections due to longer open freshwater seasons and to damage to community sanitation and drinking water infrastructure from thawing permafrost or extreme weather events |
| Pathway 3 Summary |
| Climate change is leading to increased thawing of ice and the level of pollutants in the water are increasing. Food is now contaminated and has diasastrous impacts on traditional food and cultural aspects of the Indigenous populations, which highly rely on these natural resources. (Hovelsrud et al. 2011) |
| Pathway 3 Illustration |
| Increased transport and industrial activity in the Arctic will increase contaminants levels, while melting ice, thawing permafrost, and degrading glacial snow may release legacy pollutants (POPs and metals) from past human activities. Erosion may expose waste dumps at abandoned military and oilfield sites, with potentially severe effects on the health of wildlife and local communities. These may negatively affect the immune system in animals and humans as well as degrade the quality of traditional/local foods. Food contamination problems may become particularly acute where permafrost ‘ice houses,’ used for local food storage become less effective due to thawing permafrost |

**Hunter, 2020**

***Health Condition: Mental health***

***Indigenous community: Aboriginal (Australia)***

| Pathway 1 Summary |
| --- |
| Neoliberal policies impact Indigenous health in two ways, firstly by medicalising consequences of the 'Indigenous disadvantage' and secondly, increased focus on the expression and presumed mediators of this disadvantage (violence, suicide, substance use, depression) (Hunter, 2020) |
| Pathway 1 Illustration |
| Medicalising the consequences of Indigenous disadvantage and the focus on its expression (substance use, violence, youth suicide) and putative mediators (substance dependence, personality disorder, depression) encouragesservice solutions – for which indications of improvement in outcomes are scant. |

**Jamieson, 2020**

***Health Condition: Oral health***

***Indigenous community: Global Indigenous***

| Pathway 1 Summary |
| --- |
| A description of the actual impact of the government policies which were intended to help Indigenous health (Jamieson, 2020) |
| Pathway 1 Illustration |
| Although a relationship of trust with the US government, through federal policies, is meant to improve the AI/AN–US government relationship via the provision of health, education and other social services, the forced assimilation into mainstream America of AI/AN people from their traditional lands and customs due to past government initiatives, sustained poverty, historical and intergenerational trauma, and increasing inequalities in health, education and justice have resulted in AI/AN peoples having much higher rates of unemployment, incarceration, alcohol use disorders, substance misuse, poor mental health, chronic diseases, infections and overall mortality compared with non-AI/AN people |
| Pathway 2 Summary |
| Effect of Systemic racism introduced by neoliberal policies on Indigenous Health (Jamieson, 2020) |
| Pathway 2 Illustration |
| Systemic racism is directly influenced by neoliberal policies, which by nature promote competition and supports groups in power Systemic racism has been defined as a covert form of racism expressed in the practice of social and political institutions. It originates in the operation of established societal forces, which are increasingly neoliberal in the five countries highlighted in this paper, and therefore receives less public condemnation than racism which is more individual-level. Systemic racism has unquantifiable impacts on educational attainment, political power and agency, health care, housing, employment, criminal justice, income and wealth, irrespective of country. Racism has a manifest impact on oral health inequities, with its impacts on Indigenous oral health inequalities having been empirically examined. A brief encapsulation of the arguments described above are presented in Table 1. |
| Pathway 3 Summary |
| Impact on Indigenous oral health by Neoliberal poilicies via Increased Disparities in Wealth (Jamieson, 2020) |
| Pathway 3 Illustration |
| Neoliberalism contributes to greater wealth inequities. This disproportionately impacts Indigenous populations who, irrespective of country (with the exception of Norway), are massively over-represented in lower income, under- and unemployment, and abject poverty statistics. Neoliberalism’s emphasis on ‘the self, for the self’ denies Indigenous holistic views of community sharing, participation and ownership; qualities that lead to strong social cohesion but which are cannot succeed in an environment of competition and global marketization. |
| Pathway 4 Summary |
| A detailed description of all the possible ways transnational corporations’ products and marketing impact the oral health and general health of Indigenous populations. (Jamieson, 2020) |
| Pathway 4 Illustration |
| Increased Dominance of Transnational Corporations Without Adequate Regulation or Oversight: Examples include the tobacco and sugar industries. Tobacco smoking rates are 70% in some Indigenous Australian communities, including among children as young as 8 years. In a national survey in the United States, the highest prevalence of nicotine dependence was observed among the AI/AN population, at 64%. The second highest prevalence, 54%, was observed among the White population. In Canada, 63% of the total Inuit Nunangat population aged 15 or older were daily cigarette smokers. Over half (54%) of First Nations adults living on reserves and in Northern communities smoked cigarettes, with two-fifths of these individuals smoking on average 11.6 cigarettes daily. This is compared to a prevalence of 15% of smokers in the general Canadian population, with only 10.8% reporting smoking daily. Consumption of sugar-sweetened beverages (SSBs) in Indigenous communities in Australia, Canada, Aotearoa/New Zealand and the United States is both high and normalised, with evidence suggesting that SSB consumption in these populations is likely to increase without legislation to limit it. Many Indigenous people do not trust governments with respect to safety of drinking water, which is frequently perceived to be undrinkable. In the United States, AI/AN populations have the lowest rates of indoor plumbing, with the Environmental Protection Agency stating that unregulated drinking water sources are the greatest public health risk on the Navajo Nation (due to high rates of uranium in the water). Because of the high prevalence of fracking on United States reservations, arsenic and other toxins have travelled via ground water to drinking water sources. In Canada, waterborne infections are more common in Indigenous communities, with gastrointestinal infections, skin problems and birth defects frequently reported. The same review reported concerns for mercury, lead, arsenic and toxic pollutants in First Nation community drinking water. Despite a 2015 election campaign promise to address the Indigenous long term drinking water crisis in First Nations reserves by March 2021, there remain 149 drinking water advisories (including short-term advisories, and not including all First Nation communities) as of February, 2020. Freely available and a ordable SSBs thus become the beverages of choice for many Indigenous persons for whom safe drinking water is simply not available. Norway is one of the countries with the highest intake of sugar and sweetened beverages, with dietary surveys showing that children and adolescents have significantly higher intakes of sugar than recommended. The increased dominance of mining companies in Norway, and the consequent loss of areas for reindeer herding has had substantial impacts on the mental health of the Sámi people. The impacts of poor mental health on poor oral health among Indigenous populations is well documented. |
| Pathway 5 Summary |
| Effect of Privatization of Health by incorporation of neoliberal policies on Indigenous Health (Jamieson, 2020) |
| Pathway 5 Illustration |
| Under the guise of free markets, powerful groups can influence access to dental care through promotion of specific dental service models. The US Department of Health and Human Services described how 36,000 Oglala Lakota in the United States were being serviced by only nine dentists. In the Navajo nation, there are 22 dental clinics serving 225,639 individuals, with a dentist to population ratio of 32.3 per 100,00. Different models of dental service provision for Indigenous groups have caused conflict between various stakeholder groups, for example Alaskan Native dental therapists (who are able to provide basic dental and preventive services for cheaper than traditional services) and the American Dental Association (ADA), which publicly claims to advocate for the oral health of all. Due to perceived threats against private dentistry, the ADA filed lawsuits against the Alaskan Native Tribal Health Consortium and each of the Alaskan Native dental therapists (who, at that time, were still training), and threatened both academic institutions and American Indian organisations with a loss of donations if they became involved with the Consortium. The negative effects of neoliberalism are thus exacerbated through increased emphasis on privatisation and shifts away from State welfare provision. |

**Jamieson, 2021**

***Health Condition: Oral health***

***Indigenous community: Global Indigenous***

| Pathway 1 Summary |
| --- |
| Increased dominance of transnational corporations without adequate regulation or oversight (Jamieson, 2021) |
| Pathway 1 Illustration |
| Transnational corporations products and marketing maniffestly impact the oral health of Indigenous populations. Many Indigenous people do not trust governements with respect to safety of drinking water, which is frequently perceived to be undrinkable. Freely available and affordable SSB's thus become the beverages of choice. |
| Pathway 2 Summary |
| Privatisation of Healthcare (Jamieson, 2021) |
| Pathway 2 Illustration |
| We see, negative effects of neoliberalism through increased emphasis on privatisation and shifts away from State welfare provision. |
| Pathway 3 Summary |
| Domination of concepts of personal autonomy and responsibility (Jamieson, 2021) |
| Pathway 3 Illustration |
| Neoliberal ideology promotes personal autonomy and responsibility, resulting in classist social derision of the lifestyles purchasing decisions and subsequent health outcomes of the poor. |

**Lawrence & Gibson, 2007**

***Health Condition: General health***

***Indigenous community: Aboriginal and Torres Strait Islander***

| Pathway 1 Summary |
| --- |
| The use of SRAs (Shared Responsibility Agreements) in place of health service provisions permits continuation of poor health (Lawrence & Gibson, 2007) |
| Pathway 1 Illustration |
| In the particular case of the Mulan agreement, the contract specifies that the Mulan community will ‘make sure kids shower everyday, wash face[s] twice a day . . . ensure that rubbish bins are at every house and that they are emptied twice each week . . . ensure that household pest control happens four times each year . . . [and] ensure that petrol sold through the store is not used for petrol sniffing’. In return, the Australian Government will ‘contribute $172,260 for the provision and installation of the fuel bowsers’ and the West Australian State Government will commit to having children’s health tested regularly, particularly for trachoma, skin infections and worm infestations (Draft Shared Responsbility Agreement 2004).Yet SRAs are not being used to provide Indigenous communities with services additional to those enjoyed by other non-Indigenous citizens. They are being used in an attempt to fill massive gaps in areas such as schooling, medical treatment, and housing. Rather than commit to large-scale (and necessary) programs of building schools, roads and health care centres, it appears that remote communities are being encouraged to enter contractual agreements for piecemeal handouts. |
| Pathway 2 Summary |
| Assumption of rural Indigenous communities as 'failed' or 'ungovernable' spaces permits continuation of poor health (Lawrence & Gibson, 2007) |
| Pathway 2 Illustration |
| Remote Aboriginal settlements 􏰀 ex-reserves once designed to confine Aboriginal people under colonial rule and civilise them through assimilation 􏰀 have occupied, and continue to hold, a peculiar position in the Australian geographical imagination. They have been understood as sites of rare (and dying) traditional Aboriginal culture, but also more frequently as ‘slums in the bush’, likened to third world villages, and enclaves of social problems (particularly domestic violence, vandalism, alcohol abuse and petrol sniffing). |

**Liddell & Kingston, 2021**

***Health Condition: Reproductive health***

***Indigenous community: Native American***

| Pathway 1 Summary |
| --- |
| Pollution, globalisation of food system and forced uptake of dominant culture political system (neo)increases reliance on processed foods which increase occurance of diet-related diseases (Liddell & Kingston, 2021) |
| Pathway 1 Illustration |
| Environmental pollution impacts Indigenous food sovereignty, where traditional food practices such as fishing and growing local foods may no longer be considered safe. This can force Indigenous communities to purchase more food, disrupting this relationship with food and their environment, while also leading to a less healthy, more processed diet, linked with the rising rates of diet-related diseases (e.g., diabetes and hypertension) seen among Indigenous populations in the US [38,39]. |
| Pathway 2 Summary |
| Polutants and toxins increased occurrence of fertility problems (Liddell & Kingston, 2021) |
| Pathway 2 Illustration |
| Fertilitry problems, endometriosis, polycystic ovary syndrome, frequent miscarriages, and complications during pregnancy/labour. "I look up a lot of these toxins that are around here. They’re linked to . . . fertility problems, which is a very prevalent problem around here, which does not seem to be the case a couple of decades ago, like my mom was an only child but my grandmother had a bunch of sisters and like all the generations before them had like tons and tons of kids. There was no problem. And I know more people now that have like tried- try and try and cannot have children." |
| Pathway 3 Summary |
| Chronic health conditions in community because of pollution/toxins (Liddell & Kingston, 2021) |
| Pathway 3 Illustration |
| You have all of this industrial stuff . . . We did not have that when we were young. That all came... I think that is why our people have cancer. They were not used to this . . . .This is what will kill... I said, “John Wayne did not live long enough to kill all of the Indians, so that’s what they want to do, finish killing the Indians. |
| Pathway 4 Summary |
| Increased occurrence of cancer (Liddell & Kingston, 2021) |
| Pathway 4 Illustration |
| My husband died of cancer . . . .I have two brothers that died of cancer and then I have my little brother that’s having cancer right now. But I think it’s-environment . . . And I say that to a lot of people, because I have a few people that had cancer, I think it’s seven . . . I think it’s coming from the shipyard. Because they sandblast, the sand’s all over. They paint, you have paint going all over . . . .Because cancer, the first one that I ever heard that had a cancer, in my family, was my grandmother, and she was 76-years-old, and that was in 1963. That was the first time that I heard of a family member had cancer. |
| Resistance Pathway 1 Summary |
| Environmental activists fighting to protect the health of tribal members (Liddell & Kingston, 2021) |
| Resistance Pathway 1 Illustration |
| Well, in my community we had an oil-field waste site . . . I was living in a toxic town . . . still do . . . .We got together, kids at the bus stop and parents would say their kids were sick with asthma and every month it’s the same thing. And then we realized they had dumped toxic waste inside of our community. So I became an environmentalist, not by choice but . . . .we fought for seven years against the oil and gas company . . . I was fighting for the rights of my community and my kids to live a normal, happy life as we knew it in a small town . . . .we watched kids with no asthma become asthmatic. We watched kids would grow up with . . . severe diarrhea, nausea, dizziness, vertigo. It’s different effects from all these chemicals, from the oil and gas industry. |

**Lucas, 2017**

***Health Condition: Westernised diet***

***Indigenous community: Q’eqchi’ Maya***

| Pathway 1 Summary |
| --- |
| Privitisation has influenced the articulation of new diets, leaving a strong mark on nutritional health (Lucas, 2017) |
| Pathway 1 Illustration |
| Guatemala has a history of encouraging foreign (European) investment over local indigenous rights to property, even granting additional benefits to those who married into the indigenous population to essentially mestizo-ize Guatemala (Grandia 2012, 36–38). How has the more recent imposition of Western norms and values (i.e., Western development ideologies regarding both neoliberal privatization and increased processed food consumption) influenced the articulation of new diets, local culture, and their effects on health and health beliefs within PGQ? As transnational forces enter PGQ, the influence of processed foods within the community may leave an indelible mark on local dietary habits, attitudes, and nutritional health (Monteiro et al. 2013, 22–25).Transnational corporations make rural communities vulnerable to global catastrophes, such as rising food prices. This makes it difficult for small-scale agricultural communities to thrive independently from global influences (De Janvry and Sadoulet 2010, 1328). Rising global food prices are most detrimental to small-scale farmers, particularly in Guatemala, because they alter both economic and cultural settings. The issue of cultural hybridization is explored below. While dietary shifts adversely affect indigenous health worldwide, they especially impact livelihood in Latin America. |

**Menton, 2021**

***Health Condition: Covid-19***

***Indigenous community: Indigenous peoples in Brazil (various communities)***

| Pathway 1 Summary |
| --- |
| Disproportionate Covid-19 suffering due to limited access healthcare (Menton, 2021) |
| Pathway 1 Illustration |
| Risk of COVID death for black Brazilians is 62% higher than for white Brazilians (Junior, 2020). In May, Fiocruz (2020) found that for white Brazilians, 28% of COVID-19 hospitalisations led to death while 48% of hospitalised indigenous Brazilians died and many more have died in their homes without being hospitalised. Indigenous peoples in Brazil have suffered disproportionately from the COVID-19 pandemic due to lim- ited access to an already precarious public health system together with continued attacks on their cul- tures, their territories and their way of life. These attacks come as part of the government’s attempts to further neoliberal development and undermine environmental and indigenous rights, taking advan- tage of what the Minister of Environment called ’a moment of calm while the press is focusing on the pandemic’. The indigenous population is exposed to greater risk through the degradation of their territo- ries, combined with a history of racist structural neglect (Milanez et al., 2019), particularly in provision (or lack thereof) of health ser- vices, which were further weakened since President Jair Bolsonaro took office in 2019. The indigenous movement has decried this ‘‘politics of extermination” - or, genocide (APIB, 2020; Silva, 2020; Milanez, 2020a). |
| Pathway 2 Summary |
| Mining of Indigenous territories considered an "essential service," development continued and miners brought Covid-19 into communities. (Menton, 2021) |
| Pathway 2 Illustration |
| The first registered cases of COVID-19 in southern Pará were in Parauapebas, where Vale S.A., a Brazilian multi-national mining company, has active mining sites. News of infected workers began to circulate at the beginning of April, while the company decided to maintain its operations, classified as ‘essential services’. On 11 April 2020 a Vale worker died of COVID-19, and a second died on 20 April. As the month went on, Parauapebas became the principal centre of the pandemic in the interior of Pará state, with almost 2000 cases and 64 deaths (Nogueira, 2020). In response to the pre- cariousness of local hospitals in the region, Vale brought high-level staff to Belo Horizonte in Minas Gerais for treatment, actions which led to extensive backlash by the press. It took a month from the second worker’s death for them to install a temporary hospital in Parauapebas, with support from Vale, to treat infected people but still without the capacity to deal with severe cases and with no intensive care unit.Within a few weeks, COVID-19 arrived in full force in the vil- lages of four different indigenous peoples of the region: Assurini, Mebengokrê-Xikrin, Gavião and Mebengokrê(Kayapó). Despite resitance from Indigenous leaders and court appeals, the government continued to allow development and leaders received death threats. |
| Resistance Pathway 1 Summary |
| Despite mandates to reduce support to activities that support Indigenous health during Covid, allies (such as Indigenist Missionary Council) continue to work to protect Indigenous rights (Menton, 2021) |
| Resistance Pathway 1 Illustration |
| In June 2020, allies in the national Senate were able to approve a bill for an Emergency Plan to Combat Coronavirus in Indigenous Peoples, Quilombolas and traditional populations (PL 1142) but in early July, Bolsonaro vetoed 16 of the measures. In August, Con- gress overruled Bolsonaro’s vetoes. However, in the meantime, hundreds of indigenous people had died of COVID and thousands more were infected. |
| Resistance Pathway 2 Summary |
| Emergence of grassroots organisations and local resistance due to the inabilit of neoliberalism to provide answers (Menton, 2021) |
| Resistance Pathway 2 Illustration |
| Within this context of irresponsibility and negligence by the State, we see the emergence of grassroots initiatives and local resistance. Kothari, Escobar, Salleh, Demaria, and Acosta (2020) ask ‘Can coronavirus save the planet?’ Confronted with the inabil- ity of neoliberalism to provide answers, they highlight initiatives that reveal the rebirth of radical democracy brought forth primar- ily by women and youth and based largely on Buen-Vivir (Gudynas, 2011). Another aspect of the analysis, in respect to indigenous peoples in the northeast, is that their invisibility to the wider Brazilian society and the state also means that the urgency of the pandemic led to autonomous actions - they knew that any external support would arrive too late, if at all. As Dinamam Tuxá of APIB said ‘‘We had to compensate for the absence of the state, they didn’t have a plat- form of action. The blockades are one action within reach of indige- nous peoples to help mitigate the impacts” (CIMI 2020). |
| Resistance Pathway 3 Summary |
| Self-isolation and food sovereignty enabled communities to avoid Covid-19 exposure (Menton, 2021) |
| Resistance Pathway 3 Illustration |
| They were able to avoid the spread of COVID until August. In part, they were successful in avoiding contamination due to the absence of illegal loggers and miners but also to their food sovereignty. The villages were able to remain distant from the cities and self-isolate until the month of June. |
| Resistance Pathway 4 Summary |
| Protests blocking access to mines demanding Covid-19 support from government (Menton, 2021) |
| Resistance Pathway 4 Illustration |
| In the second half of August, the Mebengokrê-Mekranoti blocked the main highway used for transportation of soya and other goods. Calling for government support for their fight against COVID and against deforestation, they refused to leave the road until the demands were met. However, that support is still missing. In late August, gunmen shot 30 bullets at the sanitation blockade set up by a Mebengokrê community in Mato Grosso. Despite the violence against them, they continue to stand firm. |

**Moore & Moore, 2021**

***Health Condition: General health (impacts of mining)***

***Indigenous community: Mayan Indigenous peoples***

| Pathway 1 Summary |
| --- |
| Neoliberal reforms to mining law opened door to foreign investment which contaminates water, land, and increases violence (Moore & Moore, 2021) |
| Pathway 1 Illustration |
| The development of the World Bank-supported Marlin mine in the northwest highlands, the first industrial mine to advance after the Peace Accords, quickly led to repression and violence against te Maya Indigenous communities who opposed its construction, health and environmental impacts from the gold and silver mine soon followed. |
| Resistance Pathway 1 Summary |
| Communities organising in peaceful resistance to protect land (Moore & Moore, 2021) |
| Resistance Pathway 1 Illustration |
| The Peaceful Resistance La Puya have defended their water and health from mining for nearly a decade, maintaining a 24-hour encampment outiside the mine entrance which continues today. Have been successful in anti-corruption cases and much of the government is now in jail or on the run. |

**Pictou, 2017**

***Health Condition: Food sovereignty***

***Indigenous community: Mi’kmaq***

| Pathway 1 Summary |
| --- |
| Under the guise of reconcilliation, unsustainable noliberal economics target Indigenous sources of food (Pictou, 2017) |
| Pathway 1 Illustration |
| Instead, under the guise of reconciliation or of building a nation-to-nation relationship, unsustainable neoliberal economics and governance models are framing treat and land-claim negotiations. In other words, the free market economy supported by Canada and states around the world targets Indigenous sources of food rooted in land-based practices, and constitutes the greatest threat to Indigenous food sovereignty. |
| Pathway 2 Summary |
| Limited funding to "close the gap" increasing poverty and hunger (Pictou, 2017) |
| Pathway 2 Illustration |
| Thus the need to "close the gap" as touted by Indigenous and non-Indigenous leadership alike, makrs a fundamental contradition to this type of (neoliberal) logic because policies aimed at closing the gap have proven to benefit only a few, while funding for social and health programs is reduced, and poverty and hunger grow. |
| Pathway 3 Summary |
| Neoliberalism perpetuates patriarchal structures that limit Indigenous women's ability to protect their Lands and Waters (Pictou, 2017) |
| Pathway 3 Illustration |
| One of the greates tragedies of this (neoliberal) approach is that it perpetuates patriarchal structures that displace and exclude Indigenous women from leadership roles, official negotiation processes, and from their own communities. In addition to protection our Lands and Water, Indigenous women across Canada continue to struggle against colonial gender discrimination conscribed by the Indian Act of 1876.The government's refusal to fully address gender inequity and the discrimination against Indigenous children, a necessary undertaking supported by several United Nations reports and Canada's own Human Rights Tribunal, while at the same time imposing neoliberal frameworks as the only way to address treaty concerns, clearly exemplifies just how deep this struggle is. |
| Resistance Pathway 1 Summary |
| Profound resilience and teachings about alternatie ways to live together with each other and wth the very source of our survivial: the natural ecosystems that sustain us (Indigenous women). (Pictou, 2017) |
| Resistance Pathway 1 Illustration |
| Indigenous women have demonstrated profound resilience and teachings about alternatie ways to live together with each other and wth the very source of our survivial: the natural ecosystems that sustain us. Therefore, Indigenous women's knowledge and experience have to be central to any decolonization process with settler-society. That includes national food-policy initiatives. Such policies cannot be just about settler systems of producing and consuming food. They also have to be about Indigenous land- and water-based practices, especially Indigenous women's practices of food and lifeways. |

**Prussing & Newbury, 2015**

***Health Condition: Health research***

***Indigenous community: Māori***

****Only resistance pathways***

| Resistance Pathway 1 Summary |
| --- |
| Māori scholars advocated and overturned NSC decision to withdraw funding for Māori research centres (Prussing & Newbury, 2015) |
| Resistance Pathway 1 Illustration |
| Smith's call for action generated an influx of support from indigenous researchers and allies around the world. Responding to this along with ongoing internal pressure, New Zealand officials then announced a reversal of the decision in May, stating that funding would support a Ma"ori CoRE e with the caveat that it would need to be reconfigured, and housed at a new location. This new turn linked restoration of funding to Ma"ori groups partici- pating in market-style competition with one another. For the next several months different groups of Ma"ori researchers worked to organize potential new sites, but through strategic reorganization as collectives. This strategy echoed Ma"ori responses to the NSC, in which an interim Kahui Ma"ori group developed to facilitate collaboration amongst different M"aori research groups interested in the three health Challenges. |

**PsychiatrNews, 1983**

***Health Condition: General health***

***Indigenous community: American Indians***

| Pathway 1 Summary |
| --- |
| Budget cuts leading to reduced health care available for Indigenous peoples (PsychiatrNews, 1983) |
| Pathway 1 Illustration |
| This legislation, Walker stressed, has led to a reduction in health acre for adopted children as well as spoises of Indians, with the elderly who have used Indian health care services all their lives suffering the most. No effort is being made to refer these people to alternative health care facilities, Walker said, and in many cases they do not have the resources to locate new facilities on their own. The third issue Walker addressed was a proposal working its way through the Reagan Administration to define the term "Indian" according to the amount of Indian blood an individual posessesses. This idea, Walker said, is yet another means of reducing the number of people who would be eligible to receive Federal health care dollars earmarked for Indians. Walker summed up APA's position by telling the subcommittee that "excluding Indian people from [Indian Health Service] funding will neither solve human nor budgetary problems. The likely result will be more Indian deaths and hospital emergency visits. The cuts proposed in urban indian health services are but another example of an inconsistent Federal policy that chooses to recognize Indian people when convenient for the government and to ignore them when the consequences of Federal action result in human suffering." |

**Ringer, 2016**

***Health Condition: General health***

***Indigenous community: Alaskan Natives/American Indians***

| Pathway 1 Summary |
| --- |
| Privitisation of fishing means fishermen are considered self-employed and have no health care insurance (Ringer, 2016) |
| Pathway 1 Illustration |
| "It’s tough, because you’re considered self-employed, so you get hit with higher taxes. Taxes are nuts. And you of course, have no health care, that’s a big part of that. It doesn’t offer a lot of stability, I think that’s why people think about settling down with someone and starting a family and it’s like, ‘well I don’t have insurance and don’t have a guaranteed job this month and what would happen? So people bail out. They don’t go into fisheries because it’s easier to get a 9-5 with insurance. (Younger local fisherman, Kodiak, 10/21/2014)" |
| Pathway 2 Summary |
| Commodification of fisheries has decreased fishing opportunities, which has a negative impact on individual and community wellbeing (Ringer, 2016) |
| Pathway 2 Illustration |
| Among maritime social scientists it is generally accepted that decreased fishing opportunities for coastal residents has a negative impact on well-being for both individuals and communities (Jones, Caveen, & Gray, 2014). Many examples around the world showcase the significance of established relationships between people and fisheries access. Coastal communities in Puerto Rico report higher levels of quality of life and well-being for those that participate in resource foraging than their non-foraging counterparts (Garcia-Quijano et al., 2015). Small-scale fishing in Mexico offers food security and income to coastal residents and yet these fishermen are considered marginalized from the governmental power structures that dictate access to fisheries (Robles-Zavala, 2014). When pathways to accessing fishing livelihoods diminish within fishing communities, community members must develop resilience strategies in order to sustain traditional livelihoods and the ability to remain in their communities (Himes- Cornell & Hoelting, 2015). Rural coastal community members in Alaska largely value both subsistence and commercial fishing activities, though there remains concern over the future with changing economies and related impacts on livelihood traditions (Holen, 2014). |
| Pathway 3 Summary |
| Decreased youth involvement in fisheries because of privitisation has contributed to increased youth addiction/substance abuse (Ringer, 2016) |
| Pathway 3 Illustration |
| Kodiak’s indigenous population and related historical or current substance abuse must also be understood in the context of post-colonialism (Hazel & Mohatt, 2001). Multigenerational trauma can be linked to high rates of substance misuse and additional related health magnitudes, whereas cultural oppression and the disempowerment of Native peoples resonates today through remnants of destabilization. Walters, Simoni, and Evans- Cambell (2002) clarified that, “The cumulative effects of these issues have been characterized as a ‘soul wound’ among American Indian peoples and constitute considerable historical trauma” (p. S109). While some respondents acknowledged varying degrees of substance abuse connected to the commercial fishing industry, Kodiak City has experienced severe spikes in drug and violence related crimes in recent years. Respondents noted the “old days” of drugs in Kodiak during the highs of the crab boom popularized cocaine, alcohol and marijuana whereas today different drugs are becoming more common within both the fishing fleet and wider community. As methamphetamines and heroin are smuggled into the region via air or ferry, addiction among youth and community residents becomes an increasing problem. Many hired skippers and captains specifically mentioned they seek crew without drug or alcohol problems when they are filling crew positions, alluding to the prevalence of addiction-related issues among potential crewmembers. There has also been intensification in thefts of guns and home burglaries in the Kodiak City area during this research signifying crime increase and potential for outbreaks of local violence. In 2014, city Chief of Police Rhonda Wallace explained to a City Council work session that, “We have guns. We have money laundering. We have violent crimes. We have drugs. We have identity theft. We have all these kinds of crimes” (Mladineo, 2014). |

**Salmon, 2011**

***Health Condition: Foetal alcohol syndrome***

***Indigenous community: Indigenous peoples in Canada***

| Pathway 1 Summary |
| --- |
| Government spending focused on FASD prevention messaging rather than health service provision (Salmon, 2011) |
| Pathway 1 Illustration |
| Contemporary neoliberal public health messaging recapitulates these obligations, emphasising women’s responsibilities to have a ‘healthy pregnancy’ and a ‘healthy baby’ by avoiding alcohol, tobacco and other drugs. As funders of maternal education campaigns targeting FASD prevention, the State’s primary interest is twofold: first, to save tax dollars spent ‘unnecessarily’ on public services for children whose disabilities are ‘entirely preventable’; and second, to promote efforts that increase the availability of workers who will contribute to the national economy without becoming ‘burdens’ or ‘drains’ on the system. Shifts towards neoliberal policy agendas over the past two decades have placed demands on nations to create ‘lean-states’ by ‘restructuring’ social programmes. These moves favour initiatives that devolve responsibilities – including responsibil- ities for ensuring the health and well-being of marginalised peoples from the State to community-based or private organisations. They also favour interventions to decrease individual ‘dependency’ on or cost to the State. These trends have informed political decisions to fund FASD prevention initiatives that rely on Aboriginal communities (rather than the State) to undertake campaigns for the prevention of FASD, on the grounds that ‘FASD births’ (i.e. the birth of people experiencing disabilities and impairments associated with maternal alcohol use) represent an 'entirely preventable' and unjustifiable 'cost to communities.' |

**Shorten, 2004**

***Health Condition: General health (inequities)***

***Indigenous community: Aboriginal and Torres Strait Islander***

| Pathway 1 Summary |
| --- |
| Low life expectancy (Shorten, 2004) |
| Pathway 1 Illustration |
| In 2004, can we say we are succeeding as a nation when Indigenous Australians cannot expect to live past their 50s…The process of change in Australia is not being managed fairly or effectively. The costs of transition in a dynamic world economy those changes fuelled by outsourcing, globalisation, deregulation - are still being borne too greatly by lower paid-workers |

**Soares, 2019**

***Health Condition: Oral health***

***Indigenous community: Guarani and Kaingang Indigenous peoples***

| Pathway 1 Summary |
| --- |
| Participation in labour market requires consumption of industrialised foods (Soares, 2019) |
| Pathway 1 Illustration |
| The Kaingang described recent changes in eating practices as a consequence of intergenerational conflicts and transformations in the group’s way of life. Participa- tion in the formal labor market and the need to follow fixed schedules require the consumption of “food of White.” Discontinuity in the practices of food cultivation was attributed to the lack of interest of the youth in traditional foods and the interference of new technologies. The emotional importance of some traditional foods (“fuá,” hunting meat)—currently prepared only in special situations—is evident. |
| Pathway 2 Summary |
| Leasing of traditional lands decreases access to traditional foods (Soares, 2019) |
| Pathway 2 Illustration |
| Although the Guarita Indigenous Land is legally recognized as a traditional terri- tory occupied by the Kaingang and Guarani peoples, there are significant land issues that affect local food production. The leasing of demarcated lands for non-Indigenous exploration, considered illegal by the Brazilian law, was reported by the Kaingang as having been occurring for decades. This phenomenon restricts access of Indigenous families to their traditional land and forms part of the long history of monetary depen- dence of the Kaingang people on outsiders. |
| Resistance Pathway 1 Summary |
| Subsistence farming to reduce reliance on industrialised products (Soares, 2019) |
| Resistance Pathway 1 Illustration |
| Among the Guarani, an understanding of the food system’s importance for the commu- nity prevails. Subsistence farming practiced by the group represents the main means of obtaining food and represents a conscious strategy of resistance and cultural reproduc- tion. The Guarani’s small crops allow the community to remain relatively independent of industrialized products and the interferences they represent: As you see the cassava there, everyone has planted. Corn also, there is plenty of corn, and it prevents us from buying food outside. |

**Sobrado, 2021**

***Health Condition: Community health***

***Indigenous community: Williche Indigenous peoples***

| Pathway 1 Summary |
| --- |
| Privitisation of health care prevented founding a health care centre until 2007 (Sobrado, 2021) |
| Pathway 1 Illustration |
| It did not have a clinic until the Padre Hurtado Center was founded in 2007 (Universidad de Los Lagos, 2011). We understand these processes as a neglect of health care (Hersch, 2013a; 2013b) linked to the privatization of health care initiated by the dictatorship and deepened by the subsequent governments, which allowed the growth of private biomedical and alternative treatment concentrated in cities (Homedes and Ugalde, 2002; 2005; Tetelboin and Salinas, 1984; Tetelboin et al., 2013). |

**Sotomayor & Barrero-Castillero, 2020**

***Health Condition: Covid-19***

***Indigenous community: Mayan Indigenous peoples***

| Pathway 1 Summary |
| --- |
| Financial constraints force many Indigenous peoples to work in larger cities in the hotel industry, which increased exposure to Covid-19 (Sotomayor & Barrero-Castillero, 2020) |
| Pathway 1 Illustration |
| Due to the financial constraints, many people from the communities go to larger cities -such as Cancun- to work in the hotel industry. Before the coronavirus-era tourism was thriving, cruise ships were coming and go- ing to and from the Riviera Maya, spring breakers were enjoying the beaches, people all around the world trav- eled to witness the natural beauty of this land. Unfortu- nately, along with their contribution to the economy, this year tourists also brought SARS-COV-2 with them. The first three confirmed cases in the area were an- nounced on March 10th, and it was until March the 30th when social distancing measures were issued, hotels were closed and people had to go back to their home- towns. And so, they took the virus with them. |
| Pathway 2 Summary |
| Limited access to healthcare heightened by Covid-19 (Sotomayor & Barrero-Castillero, 2020) |
| Pathway 2 Illustration |
| In terms of healthcare access; small community clinics frequently under-stocked with intermittent and limited medical staff, have been even more susceptible to workforce and material shortages due to the fact that medical personnel are recruited to assist in front lines at local hospitals. |
| Pathway 3 Summary |
| Lack of pandemic response that reflects social, cultural, and historical context of preexisting health disparities. (Sotomayor & Barrero-Castillero, 2020) |
| Pathway 3 Illustration |
| Another major problem- often forgotten in a global- ized world- is the cultural context. Some of these com- munities are still very rooted in their Mayan traditions that sometimes clash with the western view of medicine and healing. Understanding the interpretation of illness, health and healthcare of the communities is crucial when setting up preparedness plans. Thoughtful consid- eration of the community context helps develop a line of communication that is appropriate for them and avoids clashing cultures. In the case of the Mayas, it is import- ant to note that life, illness and health are interrelated events, and they have a direct relation with their gods and their ancestors. Life is interrelated with the physical world and the gods from the sky, earth, and underworld. This interconnectedness is reflected in the Mayan view of illness [7]. When it comes to healthcare choices, Mayans have a communitarian approach where a deci- sion is not taken autonomously by one individual, but rather as a communal decision where the extended family and the H-men (Mayan spiritual healer) participate [8]. Mental reasoning is not taken into consideration when making healthcare choices, because it is believed that the human heart is the receptor of the divine essence that comes from the “Heart of the Sky” and the “Heart of the Earth”, therefore it is only the heart that enables people to use their good sense and not the brain [9]. In this case, understanding their views on health, illness and healthcare and including the H-man in the planning and implementation process of prevention and mitigation strategies is crucial to gain the trust of the community. Building bridges of communication and trust between the leaders of our native communities is key to be able to protect these communities and to improve their ad- herence to societal guidelines. However, in a crisis there is no time to build those bridges. With this unfortunate event, one thing is clear, globalization has unintended health risks, and marginal- ized communities are left in an even more vulnerable position. |

**Stavig, 2021**

***Health Condition: Sterilisation***

***Indigenous community: Peruvian Indigenous peoples***

| Pathway 1 Summary |
| --- |
| Forced sterilization of Indigenous women to decrease poverty and increase economy (Stavig, 2021) |
| Pathway 1 Illustration |
| Held at the margins of rights, Indigenous women were then called upon as citizens (instru- mentalized) to regulate their ‘overly fecund’ bodies to power the nation’s desired poverty reduction; but as their inclusion was (and is) marked by an exclusion from full rights, Indigenous women were not asked, but forced to limit their fertility through sterilization. |

**Stephenson & Stephenson, 2016**

***Health Condition: Asthma***

***Indigenous community: Haisla First Nation***

| Pathway 1 Summary |
| --- |
| Industrial development of culturally inappropriate homes with inexpensive materials led to increased mold exposure (Stephenson & Stephenson, 2016) |
| Pathway 1 Illustration |
| The Kitamaat Band Council of the Haisla First Nation surveyed their community and informed us that 41 of 192 homes (21%) contained visible mold (MacTavish et al. 2012); additional internal mold may not be visible. The growth of mold in poorly ventilated, densely occupied houses can be rapid and represents multiple health risks including respiratory distress, vulnerability to infection by other agents, immune system disruption, cognitive problems (poor concentration) fatigue, and asthma. These are highly age related, generally affecting children more than adults (Optis et al. 2012). |

**Stienstra, 2018**

***Health Condition: Disabilities***

***Indigenous community: Indigenous peoples in Canada***

| Pathway 1 Summary |
| --- |
| Public perception about disability and access to euthanasia incresing desire to die among those with a disability (Stienstra, 2018) |
| Pathway 1 Illustration |
| These ideas about disability reflect broader neoliberal thinking, including what some have called neoliberal-ableism [2,50], which promotes inclusion and diversity while cutting social programs and failing to address the material effects of these cuts on those who rely on these programs. In addition, as Wendy Brown argues, neoliberalism applies its logic for the individual as self-investing and being “responsible for our success or failure, condemned for dependency or expectations of entitlements” [51] (p. 10). This, in turn, leads to a willingness to sacrifice one’s self to death when a citizen perceives or is told they are no longer productive or when the costs of dependency are perceived as too high [4]. In this terrain of ideas, human rights often become window dressing for the pervasive logic of neoliberal-ableism and sacrificial citizens. |
| Pathway 2 Summary |
| Neoliberal policies eroded mental health infrastructure and income assistance (Stienstra, 2018) |
| Pathway 2 Illustration |
| Indigenous boys and girls were disproportionately represented among those who were sterilized [63], and many other Indigenous children were sent to residential schools where they experienced sexual and physical abuse. Governments have only in the last decade recognized, apologized, and provided compensation to those who lived in these institutions. As Wilton [64] argues, those who lived in psychiatric institutions and were part of the de-institutionalization process of the 1980s now face more responsibility with less control over their lives. He suggests that neoliberal practices restructured the mental health and income assistance systems and eroded their funding with the result of a “return to a more traditional emphasis on professional control, justified in part through appeals to the need for increased public safety” [64] (p. 383). |
| Pathway 3 Summary |
| Commodification of care does not reflect Indigenous understandings of disability care (Stienstra, 2018) |
| Pathway 3 Illustration |
| Indigenous children with disabilities and their families face a gap between Indigenous values and approaches to both disability and childhood and what government and policy makers define as disability [39] that translates into unequal treatment. This colonial gap also relies upon reproductive relationships between Indigenous parents (most often mothers) and their children to sustain these children in the face of the disparate funding and service provisions. It also relies on the commodification of care—paying for the services of professional care providers while not recognizing the value of the mother’s care [39]. |

**Susana Ramirez, 2014**

***Health Condition: General Health***

***Indigenous community: Mbyá-Guaraní from Argentina and Chimanes, Moxeños y Yuracarés Idigenous People from Bolivia***

| Pathway 1 Summary |
| --- |
| Construction of transoceanic roads leads to irreversible damage to traditional lands (Susana Ramirez, 2014) |
| Pathway 1 Illustration |
| The Peoples living in the areas which would be affected by the construction of the roads were deemed as living under the limit of poverty by the federal government, which then argued that the construction of such roads would “save them from poverty”. Nevertheless, the previous experience with the construction of such roads is quite the opposite: deforestation plantations in monoculture and irreversible damage to ecosystems. Furthermore, the development of such areas would mean that traditional medicine would once again be incorporated by primary health services which are controlled by the government. |
| Pathway 2 Summary |
| Deterioration of the environment caused by pesticides, residuals from paper factories, contamination of water (Susana Ramirez, 2014) |
| Pathway 2 Illustration |
| The communities are no longer able to perform their traditional hunting and fishing practices and start to rely on government subsidies and tourism to survive. This increases the power that the State has over these communities. Tourism is often seen as politically neutral, but it is the front door to the political and economic system that is progressively destroying the planet. |
| Pathway 3 Summary |
| The use of bilingual interpreters/mediators for negotiations between Indigenous Peoples and the government can have severe consequences to the structure of Indigenous communities (Susana Ramirez, 2014). |
| Pathway 3 Illustration |
| The majority of the time, these individuals are not Indigenous peoples’ political leaders. The creation of “new leaders” destabilizes Indigenous communities and generates a “crisis of representation”. Such leaders often receive benefits from the government. The consequences of such external influences results in spiritual leaders taking over other non-political responsibilities |
| Pathway 4 Summary |
| The construction of reservoirs and dams in the city of Misiones, Argentina introduced multiple diseases (Susana Ramirez, 2014) |
| Pathway 4 Illustration |
| It affected the lives of 50,000 Indigenous peoples and favoured the development of multiple diseases that were not present in these areas including schistosomiasis, dengue, malaria, yellow fever, parasites, allergies, skin diseases, to name a few |
| Pathway 5 Summary |
| The cultural meaning of land destruction impacts mental wellbeing and leads to increased use of alcohol, drugs and suicide rates (Susana Ramirez, 2014) |
| Pathway 5 Illustration |
| The destruction of the environment Indigenous People live in not only causes diseases related to the destruction itself, but to the “cultural meaning” of such destruction. This results in malnutrition, alcoholism, suicide, drug addiction and others. |

**US Fed News, 2007**

***Health Condition: Child health***

***Indigenous community: Amazonian Indigenous peoples***

| Pathway 1 Summary |
| --- |
| Globalization threatens Indigenous knowledge related to poorer child health outcomes (US Fed News, 2007) |
| Pathway 1 Illustration |
| "While globalization presents many opportunites for people around the world, the loss of adaptive cultural resources for protecting health may come at a significant cost" …. Strong associations of maternal knowledge with child health (indicators include blood marker of immune function, skin-fold thickness and height to indicate growth and nutritional status) |

**Warbrick, 2016**

***Health Condition: Body weight***

***Indigenous community: Māori***

| Pathway 1 Summary |
| --- |
| Prescription of weight loss, complicit with neoliberal biopolitics, disempowers individauls when unachievable (Warbrick, 2016) |
| Pathway 1 Illustration |
| The prescription that generally follows is weight loss, something that critical health scholars and some scien- tists know is at best extremely difficult and at worst close to impossible to maintain (Aphramor, 2005; Gluckman & Hanson, 2012). This prescription of weight loss is derived from a mainstream science that has become complicit with a neoliberal biopoli- tics of the body. In New Zealand, and indeed throughout most developed countries, weight loss is seen as not only entirely reasonable, but in ‘fact’ the only ethical position that an overweight or obese person can inhabit (Dickson, 2014). This moral frame has achieved sensational success as a discourse in society, spread by the media, the health industry and perhaps more insidiously by the capital goals of the wider diet industry (Dickson, 2011) as they attempt to produce docile consumers. Although Māori are familiar with this moral frame, having been portrayed negatively in media for genera- tions (Burrows, 2009), the impact of weight loss messages has had less attention. |
| Pathway 2 Summary |
| Neoliberalism sanitised the holistic view of Māori health (Warbrick, 2016) |
| Pathway 2 Illustration |
| The confluence of these three elements – the governmental techniques of advanced liberalism, the political ideology of neoliberalism and the post-colonial attitude of bicul- turalism – produced an approach to Māori health, a biopolitics, that simultaneously sanitised the holistic view of health held by Māori ancestry, individualised and responsi- bilised Māori regarding their own health, and collected them together in an aggregate fashion as a distinctive group whose comparison to the rest of the population could then be folded back onto them in a disciplinary manner. The individualisation of health comes together with the need of some ‘measure’ of health, and the measure that has emerged as dominant in this biopolitical paradigm is weight (Guthman, 2009; LeBesco, 2011; Powell & Gard, 2014; Shannon, 2014; Warin, 2011). In New Zealand, many media and public health campaigns focus on increasing physical activity and improving diet. While initial impressions highlight the focus on enhanced lifestyle habits, rather than focusing ini- tially on weight, public health sponsored initiatives almost always include weight loss as a primary outcome measure; the primary measure of ‘success’. Thus, how does the Green Prescription6 patient who has spent 10 weeks successfully developing a habit of regular physical activity feel when the scales suggest they haven’t really achieved any- thing at all (according to weight standards)? In reality, the only option available to those with ‘Western lifestyle’ illnesses is initiatives targeted at weight loss. |
| Resistance Pathway 1 Summary |
| Māori health movement in response to rise in neoliberalism (Warbrick, 2016) |
| Resistance Pathway 1 Illustration |
| Resources could be redirected and captured for a range of pur- poses, so long as they could be premised on helping to ameliorate the problem of Māori health. For example, where once Māori health research was basically research done on ‘Maoris’ by non-Māori researchers, there are now multiple levels of health research fund- ing dedicated and set aside by the Health Research Council of New Zealand, specifically for Māori-led and Kaupapa Māori-driven research. These include studentships, masters and doctoral scholarships, and research grants for community-based research, research development, emerging researchers, full projects and research programmes, all dedicated to Māori research and Māori researchers.4 |

**Yashadhana, 2021**

***Health Condition: Eye health***

***Indigenous community: Aboriginal and Torres Strait Islander***

| Pathway 1 Summary |
| --- |
| Biomedical neoliberal language leads to communication breakdowns and exclusion from eye health decision making (Yashadhana, 2021) |
| Pathway 1 Illustration |
| In these communities, where low fundamental English literacy exists, patients described biomedical language as frightening and confusing, leading to communication breakdowns, and resulting in exclusion from eye health knowledge and decision making. ‘Some of them [clinicians] speak very hard and try to tell us with frightening words. They don’t explain things properly to us. I said to one doctor one time, please explain that more simple. So I can un- derstand properly.’ (Female patient, Community B). Clinician perceptions highlighted assumed scientific literacy profciency among patients, and reinfored biomedical 'compliance.' ‘The doctor they frighten you too, it’s how they talk you know?’ (Male patient, Community B) |
| Pathway 2 Summary |
| ACCHS clinicians upholding neoliberal values that don't align with Aboriginal cultural systems(Yashadhana, 2021) |
| Pathway 2 Illustration |
| Perspectives among clinicians diverged reflecting difference in habitus. Some saw the need for culturally responsive, empathetic service delivery (acknowledging their role as actors within the structures of the health system), while others emphasised individual patient responsibility and prioritisation of one’s health. The latter reflects the structural ideals of neoliberalism, which seeks to reduce the demand for social protection through the societal reinforcement of an individualistic moral (Bourdieu and Wacquant, 2001). However, axiologically neoliberalism is at odds with Aboriginal cultural systems, which seek to preserve social protection through family and communitarian values and practices; presenting a tension between the two value systems (doxa). ‘I think some of them are just slack to make the appointments, you know. They know all about it, but they’re slack. It’s low down on the list of priorities. They just see it as a big disruption to their, you know, lifestyle.’ (Clinician, Community A) ‘I think the barrier comes in the expectation that some clients think it’s our job to remind them of when this and that is due. There has become a system where there is no ownership on the client to take control of their own health needs.’ (Clinician, Community C) |
| Pathway 3 Summary |
| Lack of clinical Aboriginal leadership due to change in funding structures (Yashadhana, 2021) |
| Pathway 3 Illustration |
| Clinicians and patients in Community D linked the lack of Aboriginal leadership in the ACCHS to the change in organisational funding structures reflecting a tension between the need to deliver culturally responsive health services (reflecting Aboriginal values and workforce), and the neoliberalisation of care (reflecting profit focused values, and a non-Aboriginal workforce). ‘There’s less flexibility ... If your sole focus is to generate as much money as you can at some stage your client and your ability to provide good patient care is going to slip.’ (Clinician, Community D) ‘Back then your driving force was your Aboriginal Health Worker. Your managers or funding bodies finalised it and you ran it. Now its driven by people who don’t know what is going on in the commu- nity. So how do they know what is best for us?’ (AHW, Community D) |
| Resistance Pathway 1 Summary |
| Aboriginal management of care strengthening Aboriginal health and limiting influence of neoliberal values on care (Yashadhana, 2021) |
| Resistance Pathway 1 Illustration |
| Participants’ accounts of providing or receiving health care revealed foundational differences (and poor compatibility) between these two cultural systems, revealing cultural tensions between the habitus of pa- tients and clinicians as key factors that enabled cultural marginalisation (see also Yashadhana et al., 2020b). ACCHSs strive to provide culturally responsive care, which involves centring Aboriginal ways of being, knowing, and doing in the provision of clinical care. This was reflective in communities A and C, which was enabled by a strong Aboriginal workforce (including an Aboriginal diabetic nurse in Community C), and Aboriginal management of programs; resulting in positive community engagement and continuity of care. ‘The good thing is we sort of know everyone in the community so we can go to their house and if they are not there then we can ask if they know where such and such are and if they are not there then they are at this house. We will do this until we can find them.’ (AHW, Com- munity C) |

**Young & Moses, 2013**

***Health Condition: Homelessness, mental health, addiction***

***Indigenous community:*** ***Inuvialuit, Gwich'in, Métis***

| Pathway 1 Summary |
| --- |
| Neoliberal roll-back policies intensified housing shortage and obstructed development of addiction and mental health services (Young & Moses, 2013) |
| Pathway 1 Illustration |
| While proving direct causal connection between homelessness, addiction, and mental health problems is difficult, the housing shortage in Inuvik and the Beaufort-Delta is a significant contributor to the problems experienced by homeless and HtH persons. Aboriginal households are four times more likely to be overcrowded (25% compared to 7%), with remote communities experiencing a higher percentage of overcrowding (First Nations Information Governance Committee, 2006). Research on housing in Nunavut also reveals a significant shortage of housing, with one in seven people living without adequate shelter (Laird, 2007). Similarly, in the NWT, housing shortages range from 33% in larger communities such as Yellowknife to over 70% in more remote communities (Northwest Territories Bureau of Statistics, 2010). Given these data, and the geographical realities of living in the Arctic, the seriousness of homelessness and the lack of services for addicted and mentally ill persons cannot be overstated. |
